# Performance, Biomechanical Demands, Injury Burden, and Health Surveillance in Wheelchair Court Sports and Selected Para Sport Cohorts: A Systematic Review

**DOI:** 10.3390/sports14090396

**Published:** 2026-09-07

**Authors:** Prashant Kumar Choudhary, Suchishrava Choudhary, Yajuvendra Singh Rajpoot, Ana Maria Vulpe, Sohom Saha, Sema Arslan Kabasakal, Gabriel Stănică Lupu, Mihaela Anghel, Nevzat Demirci, Mihai Adrian Sava, Cristina Elena Stoica, Cristian Corneliu Drăgoi

**Affiliations:** 1 Department of Physical Education, Mahatma Gandhi Government College, Pondicherry University, North and Middle Andaman, Pondicherry 744204, India; prashantlnipe2014@gmail.com; 2 Department of Physical Education Pedagogy, Lakshmibai National Institute of Physical Education, Gwalior 474002, India; suchishrava05@gmail.com; 3 Department of Sports Management & Coaching, Lakshmibai National Institute of Physical Education, Gwalior 474002, India; yajupitu25@gmail.com; 4 Department of Physical Education and Sports Performance, “Vasile Alecsandri” University of Bacău, 600115 Bacau, Romania; sava.adrian@ub.ro (M.A.S.); cristi_dragoi@ub.ro (C.C.D.); 5 Department of Sports Psychology, Lakshmibai National Institute of Physical Education, Gwalior 474002, India; sohomsaha77@gmail.com; 6 Department of Sports Health Sciences, Yalova University Faculty of Sports Sciences, Yalova 77200, Turkey; sema.kabasakal@yalova.edu.tr; 7 Department of Physical and Occupational Therapy, “Vasile Alecsandri” University of Bacău, 600115 Bacau, Romania; anghel.mihaela@ub.ro (M.A.); cristina.popa@ub.ro (C.E.S.); 8 Department of Coaching Education, Mersin University, Mersin 33110, Turkey; ndemirci@mersin.edu.tr

**Keywords:** adaptive sport, para sport, wheelchair mobility, biomechanical load, injury surveillance, compensatory movement

## Abstract

**Background:** Research examining performance, biomechanics, workload, injury, and health in Para sport is heterogeneous and is particularly focused on wheelchair-based sports. This systematic review synthesized quantitative evidence focused predominantly on wheelchair basketball, wheelchair rugby, and wheelchair tennis, with additional evidence from Para-cycling, mixed wheelchair-sport populations, and broader Paralympic cohorts. Methods: Following the Preferred Reporting Items for Systematic Reviews and Meta-Analyses (PRISMA) 2020 guidance and predefined eligibility criteria, PubMed/MEDLINE, Scopus, Web of Science Core Collection, and Google Scholar were searched without publication-date restrictions. Owing to substantial heterogeneity in study designs, populations, sports, measurements, and outcomes, findings were synthesized narratively. Risk of bias was evaluated using design-specific appraisal tools. **Results:** Twenty-two quantitative studies were included. Evidence was focused on wheelchair basketball, wheelchair rugby, and wheelchair tennis, with additional evidence from Para-cycling, mixed wheelchair-sport populations, and broader Paralympic cohorts. Findings indicated associations among trunk function, functional classification, wheelchair mobility, upper-body function, propulsion characteristics, workload responses, and sport-specific performance. In one small study involving 10 wheelchair tennis athletes, medicine-ball throw performance was strongly correlated with serve accuracy (*r* = 0.874); this finding should not be generalized beyond that study. Prospective surveillance studies also identified meaningful injury and illness burdens across Para athlete populations. **Conclusions:** Available evidence suggests that performance and health in the represented Para sport populations are influenced by interacting functional, biomechanical, workload, and health-related factors. However, the evidence base is limited by small samples, heterogeneous methods, and a predominance of observational designs. Individualized assessment, workload monitoring, and health surveillance therefore represent provisional practice considerations rather than established best-practice recommendations.

## 1. Introduction

Adaptive and Para sport have evolved into performance-oriented, science-informed sporting environments rather than being viewed solely through a rehabilitation-focused physical activity lens. Contemporary Para athletes train and compete within systems that require physiological, biomechanical, technical, and medical support appropriate to sport-specific and impairment-related demands [1,2,3]. However, compared to able-bodied sport, adaptive sport performance is also affected by the type of impairment, functional classification, assistive equipment, wheelchair configuration, compensatory movement strategies and the interaction between the demands of the sport and the individual’s functional capacity [4,5]. It is therefore important to develop a multidimensional approach to understanding performance and health in adaptive sport, which takes into account the athlete and the adaptive sporting environment.

The upper limbs and trunk biomechanics and physiology are required to deal with significant demands in wheelchair-based sports like wheelchair basketball, wheelchair rugby and wheelchair tennis, due to the need to use the arms for wheelchair propulsion, braking, turning, acceleration, rapid changes in direction, ball/racket handling and sport-specific technical movements. In these sports, upper-body strength, shoulder function, trunk control, and harmonious arm-trunk movement are key to achieving mobility performance, executing skill, and preventing injury [6,7,8,9,10]. Body composition may also contribute to performance capacity in wheelchair athletes, as the amount and distribution of active muscle or lean mass can influence upper-body force production, power generation, and the ability to sustain repeated propulsion and sport-specific actions. However, the contribution of muscle mass to performance should be interpreted in relation to impairment characteristics, functional classification, and the musculature available for sport-specific movement 9 []. In these sports, the shoulder complex, trunk and upper extremity are the dominant sources of mobility and force production rather than the lower extremity, which plays a key role in able-bodied sport. Collectively, these studies suggest that optimizing sport performance and maintaining athlete health are closely related in wheelchair-based Para sport because the same upper-limb structures contribute to wheelchair propulsion, transfers, daily mobility, and sport-specific technical actions [10,11]. Likewise, research on wheelchair basketball has found that classification, client physique, level of play, and field position may affect performance-related outcomes, suggesting a strong relationship between functional capacity and sport role to mobility and game performance [12,13,14].

The other key factors affecting adaptive sport performance are biomechanical efficiency. Propulsion of a wheelchair is more than a locomotor activity; it is a repetitive high-load movement pattern that involves timing of the pushes, forces applied, stabilization of the trunk, and handling the wheelchair. Propulsion mechanics have been investigated using kinematic analysis, sprint testing and training-based assessment, revealing that it is possible to modify propulsion strategy, symmetry and movement control in response to sport-specific training and fatigue conditions [15,16,17]. Additionally, the chair needs to be maneuvered for stroke delivery in wheelchair tennis, and propulsion and maneuverability are further complicated by the need to control the racket during acceleration/deceleration, rotation and positioning of the chair for stroke delivery. The most recent studies have focused on wheelchair mobility field tests, push frequency, stroke time, biomechanics of the trunk and shoulders, and vibration exposure, as well as physical determinants related to serve production in wheelchair tennis players [18,19,20,21]. The results of these studies indicate that the technical skill execution and the ability to integrate it with wheelchair mobility are key factors in sport-specific performance in adaptive sport.

In addition to performance challenges, adaptive and Para sport athletes experience a significant musculoskeletal burden of stress, injury, illness and health-related interruptions. More recent prospective surveillance data from elite Para athletes has revealed a high prevalence of health issues throughout the entire season, accounting for significant training and competition disruptions both from injuries and illnesses [22,23]. Unlike most other sports, the upper limb, especially the shoulder, is constantly subjected to propulsion, transfers, sport-specific impacts and daily wheelchairs usage, making overuse symptoms and pain very relevant concerns of wheelchair athletes. This is particularly relevant as wheelchair propulsion and sport-specific wheelchair activities put repeated mechanical loading on the shoulder complex, and transfer and daily mobility activities may also further increase cumulative overuse loading [24,25,26]. In wheelchair basketball, shoulder pain has been reported to affect sport skills; meanwhile, musculoskeletal injuries have been reported in wheelchair athletes in various sport environments [27,28]. Shoulder injury prevention programs and upper limb functional rehabilitation in wheelchair basketball players have also been investigated with interventions and rehabilitation studies, focusing on the importance of addressing mobility, strength, kinematics and pain-related function in wheelchair-specific situations [29,30]. The results of this study show that the abilities to improve performance and play safely are not distinct subproblems in adaptive sport as the same upper-limb structures serve sport performance and daily independence.

Training load and health surveillance are also significant factors in Para sport, as the athlete may be susceptible to different physiological responses depending on the type of impairment, autonomic function, classification, training history and demands of the sport. Recent studies in Para athletes have suggested that the burden of injury and illness differs across sports, exposures, and impairment-related factors, and autonomic and cardiovascular regulation may also relate to the development of fatigue, recovery, and adaptation to training in athletes with neurological impairments and/or spinal cord injury (SCI) [31,32,33,34,35]. The relationship between internal and external training load in wheelchair rugby and wheelchair basketball, heart-rate and perceived-exertion methods, changes in physical condition throughout the season, and demands of the game situation have been examined in wheelchair rugby and wheelchair basketball [14,36,37,38]. But, the relationship between external workload and internal response may not be consistent among athletes, particularly if there are differences in impairment that affect the cardiovascular response, fatigue perception or movement efficiency. In addition, prospective studies have demonstrated that Paralympic athletes sustain injuries and illnesses during both training and competition time, which further emphasizes the importance of systematic surveillance of athlete health and sport-specific surveillance frameworks [22,32,39,40]. Further epidemiological evidence from Olympic, Youth Olympic and Paralympic settings corroborate the significance of understanding injury and illness patterns among elite Para athletes [41].

Although a large number of studies have been conducted, the evidence is still scattered across sport, outcomes, and study design. There is a range of studies that are focused on propulsion biomechanics, training load, shoulder pain, injury surveillance, wheelchair mobility, classification or sport-specific performance. Some reviews have focused on selected biomechanical load aspects of trunk impairment, classification, adaptive sport health, or even on psychosocial and pathophysiological measures in adaptive and Para sport athletes, but a unified synthesis that integrates all biomechanical load, injury risk, compensatory movement patterns, workload responses, functional performance, and health-surveillance components remains needed [1,11]. This gap is significant given that coaches, clinicians, classifiers, sport scientists and administrators need a unified knowledge of how performance demands and health risks interrelate in the context of adaptive sport.

Accordingly, the primary review question was: What quantitative evidence describes the relationships among sport-specific performance, biomechanical and wheelchair-related demands, workload exposure, injury and illness burden, and health surveillance in wheelchair court sports and selected Para sport cohorts? The secondary objectives were to: (i) characterize the sports, athlete populations, and study designs represented in the available literature; (ii) synthesize evidence concerning wheelchair mobility, trunk function, functional classification, propulsion mechanics, upper-limb demands, and internal and external workload; (iii) summarize injury, illness, musculoskeletal pain, and health-surveillance findings; and (iv) identify methodological limitations and evidence gaps that should guide future Para sport research.

## 2. Materials and Methods

### 2.1. Eligibility Criteria

Eligibility criteria were formulated using the Population, Intervention/Exposure, Comparison, and Outcome (PICO) framework and followed the PRISMA 2020 guidance (Supplementary Materials) for transparent reporting of systematic reviews [42]. Competitive adaptive sport and Para sport athletes were included in the population as these groups additionally use impairment-specific classification, sport-specific loading, and different performance demands [1,4,11]. Only trained, elite, collegiate, junior, national, or international athlete studies involving structured training, competition, simulated sports tasks, or sport-specific testing were included in the study. To ensure population specificity, studies that included only one of the following groups—able-bodied athletes, rehabilitation patients, sedentary individuals, or wheelchair users who were not involved in organized sporting activities—were omitted [1,5]. The following exposures were selected as they are frequently explored in Para sport health and performance research [37,38,43]: sport participation, training load, match play, wheelchair propulsion, wheelchair functional classification, wheelchair biomechanical testing, wheelchair workload monitoring, injury-prevention programs, competitive-season exposure and wheelchair set-up testing. Single-group quantitative studies were also considered if relevant numerical data was reported, since Para sport research may be conducted in small and very specific sporting samples [1,40]. Biomechanical-, internal- and external-load variables, injury risk, musculoskeletal pain, health problems, wheelchair propulsion mechanics, functional performance, range of motion, strength, asymmetry, heart rate, rating of perceived exertion, player load, sprint performance, injury incidence, illness incidence and injury or illness prevalence were all considered as outcomes [37,40,41]. Studies had to provide quantitative data that could be extracted and presented as mean ± SD, *p* values, correlations, effect sizes, incidence rates, prevalence values, odds ratios, hazard ratios or injury burden. Reviews, systematic reviews, meta-analyses, bibliometric studies, editorials, commentaries, case reports, protocols, conference abstracts with limited information and qualitative-only studies were not included. No publication-date restriction was applied. Eligibility was determined solely according to the prespecified population, sport context, exposure, outcome, study design, publication type, language, and quantitative data criteria. Consequently, no otherwise eligible study was excluded solely because of its publication year. The concentration of included studies in more recent years reflects the available evidence rather than a prespecified preference for recent publications [1,22,38,41]. This systematic review was conducted in accordance with the PRISMA 2020 reporting guidance. The review protocol was not prospectively registered.

### 2.2. Information Sources and Search Strategy

A systematic and replicable literature review was performed to identify quantitative studies investigating exercise exposure, biomechanical loading, risk of injury and compensatory movement patterns in adaptive sport and Para sport populations. The following electronic databases were searched: PubMed/MEDLINE, Scopus, Web of Science Core Collection and Google Scholar. For Google Scholar, because complex Boolean searches are not consistently supported, multiple simplified keyword searches were performed. Google Scholar was last searched on 16 July 2026. For each search combination, the first 200 results were screened in the order returned by Google Scholar using its default relevance ranking. Records retrieved through Google Scholar were cross-checked against those identified through PubMed/MEDLINE, Scopus, and Web of Science Core Collection. Duplicate records were identified using the digital object identifier (DOI) where available and, otherwise, by matching article title, first author, publication year, and journal; unique records proceeded to screening. The following electronic databases were searched: PubMed/MEDLINE, Scopus, Web of Science Core Collection, and Google Scholar. The final searches were conducted between 13 and 16 July 2026: PubMed/MEDLINE was last searched on 13 July 2026, Scopus on 14 July 2026, Web of Science Core Collection on 15 July 2026, and Google Scholar on 16 July 2026. These dates represent the final searches performed before completion of study selection and synthesis. The databases were selected because they provide broad coverage of sports science, rehabilitation, biomechanics, exercise physiology, disability sport, Para sport, injury epidemiology, and health-surveillance research. The search strategy was formulated based on the PICO format and integrated four main concepts: adaptive sport population, exercise or training exposure, biomechanical or workload variables and injury or compensatory motion outcomes. Boolean, phrases, truncation and database-specific field tags were employed as appropriate. There was no restriction on the publication date of the articles, but only full-text articles in English published by peer-reviewed journals were selected.

For PubMed/MEDLINE, the following search string was used: ((“adaptive sport” OR “adaptive sports” OR “Para sport” OR “Para sports” OR “Paralympic” OR “Paralympic athletes” OR “wheelchair sport” OR “wheelchair athletes” OR “wheelchair basketball” OR “wheelchair rugby” OR “wheelchair tennis” OR “Para-cycling” OR “disability sport”) AND (“exercise exposure” OR “training exposure” OR “training load” OR “match load” OR “competition load” OR “external load” OR “internal load” OR “workload” OR “player load” OR “sport participation” OR “wheelchair propulsion” OR “propulsion biomechanics” OR “functional classification”) AND (“biomechanical load” OR “biomechanics” OR “kinematics” OR “kinetics” OR “asymmetry” OR “movement pattern” OR “compensatory movement” OR “shoulder pain” OR “musculoskeletal injury” OR “injury” OR “injuries” OR “illness” OR “health problems” OR “injury risk”) AND (“quantitative” OR “experimental” OR “intervention” OR “cross-sectional” OR “cohort” OR “observational” OR “pre-post” OR “prospective”)) NOT (“systematic review” OR “meta-analysis” OR “bibliometric” OR “protocol” OR “qualitative” OR “editorial” OR “commentary”). For Scopus, the search was adapted using the TITLE-ABS-KEY field as follows: TITLE-ABS-KEY ((“adaptive sport” OR “adaptive sports” OR “Para sport” OR “Para sports” OR “Paralympic” OR “Paralympic athletes” OR “wheelchair sport” OR “wheelchair athletes” OR “wheelchair basketball” OR “wheelchair rugby” OR “wheelchair tennis” OR “Para-cycling” OR “disability sport”) AND (“exercise exposure” OR “training exposure” OR “training load” OR “match load” OR “competition load” OR “external load” OR “internal load” OR “workload” OR “player load” OR “sport participation” OR “wheelchair propulsion” OR “propulsion biomechanics” OR “functional classification”) AND (“biomechanical load” OR “biomechanics” OR “kinematics” OR “kinetics” OR “asymmetry” OR “movement pattern” OR “compensatory movement” OR “shoulder pain” OR “musculoskeletal injury” OR “injury” OR “injuries” OR “illness” OR “health problems” OR “injury risk”) AND (“quantitative” OR “experimental” OR “intervention” OR “cross-sectional” OR “cohort” OR “observational” OR “pre-post” OR “prospective”)) AND NOT TITLE-ABS-KEY (“systematic review” OR “meta-analysis” OR “bibliometric” OR “protocol” OR “qualitative” OR “editorial” OR “commentary”). For Web of Science Core Collection, the search was adapted using the topic field: TS = ((“adaptive sport” OR “adaptive sports” OR “Para sport” OR “Para sports” OR “Paralympic” OR “Paralympic athletes” OR “wheelchair sport” OR “wheelchair athletes” OR “wheelchair basketball” OR “wheelchair rugby” OR “wheelchair tennis” OR “Para-cycling” OR “disability sport”) AND (“exercise exposure” OR “training exposure” OR “training load” OR “match load” OR “competition load” OR “external load” OR “internal load” OR “workload” OR “player load” OR “sport participation” OR “wheelchair propulsion” OR “propulsion biomechanics” OR “functional classification”) AND (“biomechanical load” OR “biomechanics” OR “kinematics” OR “kinetics” OR “asymmetry” OR “movement pattern” OR “compensatory movement” OR “shoulder pain” OR “musculoskeletal injury” OR “injury” OR “injuries” OR “illness” OR “health problems” OR “injury risk”) AND (“quantitative” OR “experimental” OR “intervention” OR “cross-sectional” OR “cohort” OR “observational” OR “pre-post” OR “prospective”)) NOT TS = (“systematic review” OR “meta-analysis” OR “bibliometric” OR “protocol” OR “qualitative” OR “editorial” OR “commentary”). For Google Scholar, because complex Boolean searches are not consistently supported, multiple simplified keyword searches were performed. The first 200 results from each relevant search combination were screened. The Google Scholar search combinations included: “wheelchair basketball” “training load” “injury risk” biomechanics; “wheelchair rugby” propulsion biomechanics injury shoulder pain; “Para sport” “health problems” injury illness prospective cohort; “Paralympic athletes” injury illness “training load”; “adaptive sport” “biomechanical load” “compensatory movement”; “wheelchair athletes” “shoulder pain” “musculoskeletal injury”; “wheelchair propulsion” “functional classification” “performance”; and “Para-cycling” injury illness surveillance athletes. Regarding the electronic database searching, the reference lists of all eligible full-text articles were manually screened to identify additional relevant studies. Citation chasing was also performed for key articles related to wheelchair basketball load monitoring, wheelchair rugby propulsion biomechanics, Para athlete injury surveillance, shoulder pain, and functional classification.

The database strategy intentionally emphasized specificity by requiring the intersection of the Para sport population, sport exposure/workload, biomechanical or health-related outcomes, and quantitative study-design terminology. This structure was selected because the review focused on quantitative evidence linking sport-specific performance and exposure with biomechanical, workload, injury, or health-related outcomes rather than attempting to catalogue every performance or epidemiological study conducted in Para sport. We acknowledge that requiring simultaneous concept blocks may reduce search sensitivity. To supplement the structured database searches, simplified domain-specific Google Scholar searches and reference-list/citation-chasing procedures were therefore used to identify potentially relevant studies that might not have been retrieved through the primary Boolean strategy. Accordingly, the present review should be interpreted as a synthesis of integrated quantitative evidence within the represented sport contexts rather than an exhaustive synthesis of all Para sport research.

### 2.3. Selection Process

The process of selecting studies was performed following the PRISMA 2020 flow chart for the identification, screening, eligibility assessment and inclusion of studies into the systematic review [42]. First, all records found by a database and register search were gathered and duplicates were removed. Titles and abstracts were independently reviewed against the predetermined eligibility criteria after the duplicate removal and after the exclusion of obviously irrelevant entries. Studies that were potentially relevant were retrieved in full text and detailed assessment was performed. Full-text screening: Each article was assessed using PICO-based inclusion and exclusion criteria. Specific attention was paid to the type of sport or Para sport athlete, type of exposure or condition (adaptive sport vs. Para sport), and types of outcomes (quantitative sport-specific, quantitative injury, quantitative compensatory movement, quantitative performance, or quantitative health surveillance). Articles were removed when they included rehabilitation populations that were not sports-related, were not relevant to the field of adaptive sports, provided only qualitative data, were review articles, or provided no numerical data from which to extract. Disagreements were resolved through discussion, with a third reviewer consulted when consensus could not be reached [44]. A total of 22 studies were chosen for the systematic review. A PRISMA flow diagram was used to summarize the complete selection process, detailing the number of records identified, screened, excluded, assessed for eligibility and eventually included.

Eligibility criteria as included in Table 1 have been updated to reflect the final inclusion of 22 quantitative studies on adaptive and Para sport athletes from wheelchair basketball, wheelchair rugby, wheelchair tennis, Para-cycling and mixed-sport athletes in the Paralympics. The review included injury risk, health surveillance, biomechanical loads and compensatory movement outcomes, and sport-specific performance and wheelchair mobility outcomes, including sprint performance, agility, serve accuracy, upper-body explosive strength, trunk function, functional classification, propulsion mechanics, internal and external loads, range of motion, strength, asymmetry, rating of perceived exertion, heart rate, player loads, and injury and illness incidence and prevalence.

The study designs included were randomized and non-randomized experimental studies, pre–post intervention studies, cross-sectional and observational studies, prospective cohort studies, longitudinal surveillance studies, correlational field-test studies, predictive modeling studies, and biomechanical testing studies as long as quantitative data that could be extracted were reported. Studies that included only able-bodied athletes, rehabilitation patients (excluding sport), or general wheelchair users, qualitative-only studies, studies that were reviews, case reports, protocols without results, or studies that did not report numerical data in the text that could be extracted were not included. The evidence base for the final was primarily from the last ten years (2015–2026) as this is the timeframe of current research related to adaptive sport performance, workload, biomechanics, injury risk, and health monitoring.

### 2.4. Data Extraction Process

A standardized data extraction form was developed to collect methodological, participant, exposure, outcome, and statistical information relevant to the review objectives. Data extraction was performed independently by two reviewers. For each included study, the following information was extracted: author(s) and year of publication, country, study design, sport and athlete population, sample characteristics, age and sex distribution, type of impairment, functional classification where applicable, training or competitive level, exposure or intervention, comparison or grouping strategy, primary outcome variables, measurement instruments, and key findings relevant to the review.

Outcome-level information included internal and external training load, heart rate, rating of perceived exertion, player load, propulsion biomechanics, wheelchair mobility and sprint performance, range of motion, strength, asymmetry, shoulder pain, injury and illness prevalence or incidence, and other performance- or health-related quantitative measures. Numerical data were extracted as reported in the original studies, including means and standard deviations, pre–post values, group comparisons, correlations, *p* values, *t* values, confidence intervals, prevalence and incidence estimates, odds ratios, hazard ratios, and other reported effect estimates where available.

After independent extraction, the two reviewers compared the completed extraction forms to identify discrepancies. Disagreements were resolved through discussion, with a third reviewer consulted when consensus could not be reached. Effect estimates reported by the original studies were retained in their original metric. No de novo standardized mean differences were calculated because the included studies differed substantially in study design, comparison structure, outcome definition, measurement scale, and availability of the statistical information required for valid standardized effect-size calculation.

### 2.5. Risk of Bias Assessment

The risk of bias was evaluated using design-specific appraisal tools, as recommended for transparent reporting of bias assessment in systematic reviews [42]. Because the included evidence comprised randomized experimental studies, non-randomized pre–post intervention studies, cross-sectional observational studies, biomechanical and load-monitoring investigations, performance-prediction studies, and prospective or longitudinal cohort-surveillance studies, no single appraisal instrument was considered appropriate for all study designs [42,45]. The Cochrane Risk of Bias 2 (RoB 2) tool was applied to randomized intervention studies. The randomized experimental pre–post study by Sterneet al. (2019) was therefore assessed using RoB 2, which considers bias arising from the randomization process, deviations from intended interventions, missing outcome data, outcome measurement, and selective reporting [15,46]. The Risk Of Bias In Non-randomized Studies of Interventions (ROBINS-I) tool was used for non-randomized intervention studies because it permits assessment of bias related to confounding, participant selection, classification of interventions, deviations from intended interventions, missing data, outcome measurement, and selection of the reported result [47].

Prospective cohort and longitudinal injury- and illness-surveillance studies were evaluated using the Joanna Briggs Institute (JBI) Critical Appraisal Checklist for Cohort Studies [48], with consideration of participant selection, exposure measurement, identification and management of confounding, outcome measurement, completeness of follow-up, and appropriateness of statistical analysis. The JBI Critical Appraisal Checklist for Analytical Cross-Sectional Studies [48] was applied to studies whose underlying design was genuinely cross-sectional, including relevant observational, prevalence, functional-classification, biomechanical, reliability, validity, and performance-assessment studies. For biomechanical modeling and performance-prediction investigations, the appraisal instrument was selected according to the underlying study design, with additional attention to measurement validity, modeling assumptions, prediction procedures, and statistical interpretation. Longitudinal or repeated-measures studies were not classified as cross-sectional solely because biomechanical, workload, or performance outcomes were evaluated.

Two reviewers independently performed the risk-of-bias assessments for all included studies. Disagreements were resolved through discussion, with a third reviewer consulted when consensus could not be reached, in accordance with recommended systematic-review methodology [42,45]. Because RoB 2, ROBINS-I, and the JBI appraisal checklists differ in their domains, response categories, and overall judgment frameworks, assessments from these instruments were interpreted within their respective methodological frameworks rather than treated as directly equivalent. For ease of visual presentation, a common traffic-light color scheme was used in the summary table only as a descriptive aid. Green represented a favorable judgment, low risk of bias, or adequate methodological reporting according to the relevant appraisal instrument; yellow represented some concerns, uncertainty, or insufficient methodological reporting; and red represented an unfavorable judgment or serious/high risk of bias within the relevant instrument. The color categories therefore do not represent mathematically or methodologically equivalent scores across RoB 2, ROBINS-I, and JBI, and no numerical pooling or cross-tool scoring of risk-of-bias judgments was undertaken. Overall assessments were interpreted with reference to the domains relevant to each study design, including participant selection or sampling, confounding, intervention/exposure or performance measurement, outcome measurement, missing data or follow-up, statistical analysis, and study-design limitations, and were used to inform interpretation of the synthesized findings [42].

### 2.6. Data Synthesis Methods

Due to methodological diversity among the included studies in terms of study design, type of sport, population characteristics, conditions of exposure, measurement tools and reported outcomes, a structured narrative synthesis was performed. Experimental, pre–post, cross-sectional, observational, biomechanical and prospective cohort designs were included in the evidence. The synthesis focused around the key thematic areas of the review: exposure and load of the exercise, biomechanical load and wheelchair propulsion mechanics, injury risk and musculoskeletal pain, functional classification and performance demands, and compensatory movement patterns or asymmetry. Studies were classified based on the type of sport, design of the study, exposure conditions, and the category of outcome. Quantitative findings were therefore summarized using the statistics reported by the original studies, including means and standard deviations, correlations, prevalence and incidence estimates, odds ratios, hazard ratios, *p* values, confidence intervals, and reported effect estimates where available. Incidence proportions, incidence rates, prevalence, hazard ratios, and injury burden were descriptively synthesized for injury- and illness-surveillance studies.

## 3. Results

Twenty-two quantitative studies were included in the present systematic review, which evaluated exercise exposure, biomechanical load, injury risk, compensatory movement patterns, performance-related outcomes, and health surveillance in adaptive and Para sport athletes. The studies included in this inventory included wheelchair basketball, wheelchair rugby, wheelchair tennis, Para-cycling and mixed groups of Paralympians. Study designs ranged from cross-sectional, observational, experimental, pre–post intervention, prospective cohort, longitudinal surveillance, correlational field-test and biomechanical modeling. In the evidence included, the major outcomes were classified into sport-specific performance measures, wheelchair movement, propulsion biomechanics, functional classification, trunk function, internal and external loads, musculoskeletal pain, injury and illness incidence, range of motion, strength, asymmetry, fatigue-related adaptations and burden of health problems. Overall, results are reported with regard to study selection, study characteristics (included studies), risk of bias, and major quantitative results for the performance, biomechanical, workload, injury, and health-related domains.

**Figure 1:**
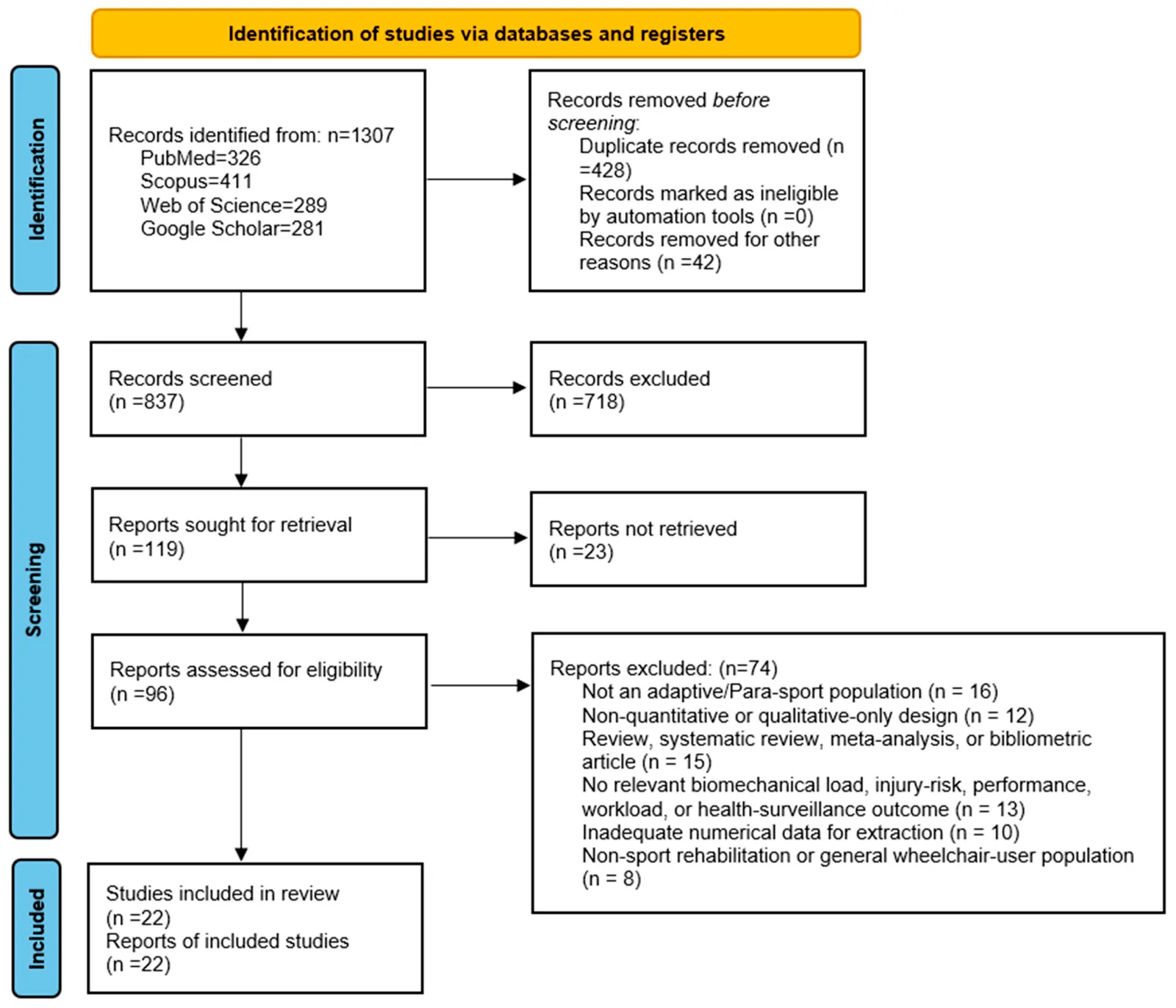
PRISMA 2020 flow diagram illustrating study identification, screening, eligibility assessment, and inclusion.

Table 2 summarizes the characteristics of the 22 quantitative studies that were included in the systematic review, including populations from adaptive and Para sport represented by wheelchair basketball, wheelchair rugby, wheelchair tennis, Para-cycling, and mixed groups of Paralympic athletes (see Figure 1). The studies included various quantitative study designs such as cross-sectional, observational, experimental, pre–post intervention, prospective cohort, longitudinal surveillance, correlational field-test, and biomechanical modeling. The samples included athletes at the junior, collegiate, national, international, elite, and professional level, with a variety of impairments, including spinal cord injury, amputation, cerebral palsy, spina bifida, musculoskeletal impairment, neurological impairment, visual impairment, and impaired muscle power.

The studies reviewed were those involving sport-specific exposures (match play, training load, wheelchair propulsion, sprint testing, agility testing, serve accuracy, functional classification, injury-prevention programs, wheelchair set-up testing, and monitoring during the competitive season). Outcomes encompassed biomechanical load, internal and external load, propulsion mechanics, functional performance, strength, range of motion, asymmetry, RPE, HR, player load, injury/illness incidence, musculoskeletal pain and sport-specific performance indicators. In conclusion, the table shows that adaptive sport research employs a variety of measurement instruments, such as IMUs, motion analysis systems, field tests, questionnaires, heart-rate monitors, dynamometers and injury-surveillance platforms. These 22 studies collectively offer a wide range of quantitative evidence on demands for performance, injury risk, compensatory movement, workload responses, and health monitoring requirements in adaptive and Para sport athletes.

A summary of the risk of bias of the 22 studies in the systematic review is provided in Table 3. It includes domain-wise judgments on selection and sampling, confounding, exposure/intervention measurement, outcome measurement, missing data, statistical analysis, and overall risk that are summarized for each study. Overall, most studies were considered to have some concerns, primarily because of small sample sizes, questionable sampling methods, inadequate control of confounding variables, and insufficient reporting of methods.

Several studies demonstrated better methodological quality regarding the exposure, intervention, or performance measurement due to the use of objective testing instruments and structured assessment procedures. A few studies, however, were found to have uncontrolled designs, confounding issues, prevalence-based limitations, or inadequate statistical analysis and therefore high or serious overall risk of bias. The cohort-surveillance studies typically demonstrated relatively high-quality statistical reporting, but some issues were noted related to sampling, outcome measurement, or missing data. Overall, the evidence base is informative; however, findings should be interpreted cautiously given the methodological limitations and risk-of-bias concerns identified across several included studies.

## 4. Discussion

The findings offer a multidimensional perspective on adaptive and Para sport performance, in which athletic performance is influenced by the interaction between functional capacity, wheelchair mobility, sport-specific skill, biomechanical efficiency, training exposure and health status. In the present review, functional capacity refers to the athlete’s impairment-related ability to generate, stabilize, and control movement during sport-specific tasks through the available contribution of trunk control, upper-limb function, strength, range of motion, and neuromuscular control. This concept is distinct from wheelchair mobility, which reflects the execution of movement with the wheelchair; sport-specific skill, which reflects the technical execution of a particular sporting task; biomechanical efficiency, which describes how movement and force are produced and transferred; training exposure, which represents the accumulated physical and sport-specific workload; and health status, which encompasses injury, illness, symptoms, fatigue, and recovery. The evidence does not illustrate a consistent and similar pattern of physical and technical demands and movement solutions among all Paralympic sport athletes but suggests each sport has its own physical and technical demands that will necessitate unique movement solutions. The upper-limb functions, trunk control, efficient propulsion, acceleration, turning and repeated high-intensity actions are emphasized in wheelchair-based sports while, in Para sport contexts with an endurance focus, the emphasis is on prolonged exposure, recovery and health monitoring. In the provided evidence, performance and health seem to be correlated closely, as the same movement strategies used for gaining speed, control, power and technical accuracy may also contribute to an increase in mechanical stress over time. Based on the themes highlighted by the findings, the discussion is structured into the following themes: sport-specific performance, functional classification, propulsion mechanics, workload responses, musculoskeletal stress, compensatory movement patterns, and athlete health burden. From an applied perspective, these findings support considering functional, technical, biomechanical, workload, and health-related characteristics together rather than interpreting any single performance domain in isolation.

### 4.1. Sport-Specific Performance and Wheelchair Mobility

Performance-related evidence in the present review focused predominantly on the wheelchair court sports of wheelchair basketball, wheelchair rugby, and wheelchair tennis. Para-cycling and mixed Paralympic cohorts contributed primarily to the injury, illness, and health-surveillance components of the synthesis and should be considered separately from wheelchair court-sport performance evidence. Across the wheelchair court-sport studies, performance was associated with the interaction of impairment-related function, wheelchair mobility, trunk control, upper-body capacity, wheelchair configuration, and sport-specific technical demands. There is evidence that the trunk impairment test is correlated with activity limitation, the upper-body strength test, wheelchair sprint capacity, acceleration, turning ability, and sport-specific mobility test, which suggests the need for multidimensional functional profiles, as opposed to isolated strength and speed measures, to assess performance in wheelchair court sports [10,11,21,52]. Using wheelchairs to serve accurate balls and generate serve velocity and propulsion velocity, as well as to move efficiently in the field, are key factors in technical execution and achieving adequate coverage of the court [10,21,52]. In a similar manner, evidence from wheelchair rugby, wheelchair basketball and Para-cycling suggests that functional capacity, trunk and upper-limb function, wheelchair configuration, anaerobic power and sport-specific technical demands are interrelated and will determine the performance outcome of the athlete [18,19,21,53,54]. These results are not to be interpreted as strength-based results. Instead, they suggest that wheelchair tennis performance is influenced by the athletes’ capacity to generate forces, to transmit the force up the trunk and upper limbs, and at the same time to control the interaction between the wheelchair and the racket in highly constrained sport tasks. Practically, athlete profiling in wheelchair court sports may therefore benefit from combining wheelchair-mobility assessment with trunk and upper-body function and sport-specific technical performance measures.

One of the key findings in the evidence included is the functional classification and trunk control’s effect on the expression of functional performance in adaptive sport athletes. For the athletes who had higher trunk control and functional capacity in wheelchair tennis and wheelchair basketball, the mobility, acceleration, rotation, and load tolerance during sport-specific tasks were higher [10,12,14]. The evidence also indicates, however, that classification is not sufficient to account for performance. Other athletes may make up for this in part with a more refined propulsion technique, optimal wheelchair configuration or increased sport-specific experience [49]. This is why classification is an important but insufficient explanatory model. The results suggest that static classification categories should not be used in isolation from dynamic performance measures, as sport performance reflects more than an individual’s level of impairment. Accordingly, functional classification should inform, rather than replace, individualized assessment of wheelchair mobility, propulsion technique, equipment configuration, and sport-specific performance.

### 4.2. Propulsion Mechanics and Movement Efficiency

The results of the propulsion-related findings indicate that the quality and efficiency of the propulsion cycle have a significant impact on wheelchair sport performance. Research on wheelchair tennis, wheelchair basketball, and wheelchair rugby has shown that higher performing athletes are able to use more efficient propulsion techniques, such as better push timing, smoother force application, improved bilateral coordination, and better wheelchair handling [15,17,19,49]. The results are of significance as they move the focus of interpretation from the outcome of the speed to the process of the movement. A significant difference in final sprint time does not necessarily indicate a sizeable improvement in biomechanics, such as push frequency, direction of force, symmetry or control of the movement. So, propulsion mechanics should be considered not only as a performance determinant but also as a potential indicator of compensatory movement behavior in adaptive sport. For coaches and sport scientists, propulsion-quality variables may therefore be considered alongside conventional performance outcomes such as sprint time and wheelchair-mobility performance. Similar observations have been reported in athletes with intellectual disabilities, where movement efficiency and neuromuscular control were strongly associated with functional performance outcomes. Dynamic balance, agility, and jumping ability were identified as important contributors to sprint performance, highlighting the close relationship between movement quality and athletic execution in adaptive populations [55]. Similar evidence from pediatric populations indicates that motor competence is closely associated with movement behaviors and cognitive functions, including inhibition and visuospatial working memory, highlighting the multidimensional role of motor development in physical performance and functional outcomes throughout childhood [56].

Furthermore, targeted neuromuscular interventions combining balance, strength, and plyometric training have been shown to improve physical performance in athletes with intellectual disabilities, emphasizing the role of integrated motor development in enhancing movement efficiency and athletic performance [57]. As this evidence is contextual rather than part of the 22-study systematic synthesis, it may help to generate hypotheses concerning movement-quality assessment but should not independently determine practice recommendations.

### 4.3. Internal- and External-Load Responses

The workload studies indicate that there are unique internal- and external-load profiles across different sports, competition settings, functional categorization and movement demands for adaptive sport athletes. Competition seems to place larger physical and physiological demands than training, especially with actions that require accelerated, decelerated, rotational, impact, and repeated high-intensity movements [14,51]. However, the relationship between external workload and internal physiological response is not necessarily linear. Subjective exertion measures can provide useful information, but they may not fully represent physiological load in athletes whose impairment characteristics, autonomic function, upper-limb fatigue responses, or movement efficiency differ [36]. Accordingly, workload should be interpreted using multiple complementary indicators rather than a single isolated measure. Beyond physiological workload, cognitive demands may also influence motor control and balance regulation. Research involving adolescents with intellectual disabilities demonstrated that variations in cognitive load and visual conditions significantly affected postural stability, suggesting that external demands should be interpreted within a broader biopsychosocial framework of adaptive sport participation [58]. Differences in impairment, classification, training experience, and mobility strategy in using wheelchairs can result in very different internal stress while two athletes perform the same external tasks in adaptive sport. In practice, workload monitoring may therefore be strengthened by combining internal and external indicators and interpreting them in relation to impairment characteristics, functional classification, training experience, and sport-specific movement demands.

### 4.4. Shoulder Pain, Upper-Limb Stress, and Musculoskeletal Demand

The results of the review support the notion that shoulder pain and upper-limb stress are key issues in the field of wheelchair-based adaptive sport. Repeated mechanical loading of the shoulder occurs in wheelchair basketball, wheelchair rugby, wheelchair tennis, and mixed wheelchair sport across a range of movements, including propulsion, braking, turning, shooting, passing, blocking, and racket-based movements [27,28,29,30]. This repeated loading is not only a sport-specific problem but is also related to the daily mobility of the wheelchair user, especially in wheelchair athletes, who are likely to have high total exposure to the upper limb. Based on the evidence, there is evidence of pain and dysfunction when the demands of performance are higher than the capacity of the shoulder to produce force repeatedly, to stabilize the shoulder, and to recover from the force production. Meanwhile, findings from interventions suggest that shoulder function could be improved even if changes in strength are minimal, through interventions focused on improving mobility, scapular control, flexibility and movement quality. This implies that, in wheelchair athletes, shoulder health should be considered with respect to how it functions as part of the whole, rather than just strength at the level of the shoulder. This holistic perspective is consistent with the broader adaptive sport literature showing that postural control, balance capacity, and movement quality are fundamental determinants of functional performance and injury resilience among individuals with disabilities [58,59]. Practically, shoulder-health monitoring may therefore extend beyond isolated strength measures to include symptoms, mobility, movement quality, cumulative upper-limb exposure, and recovery status.

### 4.5. Fatigue and Compensatory Movement Patterns

One of the recurring themes within the included evidence was the use of task-specific movement adaptations in response to impairment-related functional constraints and sport-specific demands. In wheelchair rugby and wheelchair tennis, athletes modified trunk position, shoulder movement, push timing, and propulsion strategies while attempting to maintain mobility, control, and technical performance under demanding conditions [16,17,20]. Such compensatory movement patterns should not necessarily be interpreted as dysfunctional. Rather, they may represent functional adaptations that enable athletes to perform sport-specific tasks within their available physical and biomechanical capacities. Nevertheless, these adaptations may also alter movement symmetry, redistribute mechanical loading across the upper extremities, and increase localized stress on particular joints or tissues. Their implications may therefore depend on the athlete’s impairment characteristics, technique, fatigue status, playing role, and cumulative exposure.

Contextual evidence outside the 22 studies included in the systematic synthesis provides additional perspective on this concept. Studies involving adolescents and other populations with intellectual disabilities have reported alterations in postural balance and movement control in response to visual perturbations, cognitive demands, and prolonged smartphone-related exposure [57,60,61]. Although these studies did not meet the eligibility criteria for the present systematic review and were not used to derive its principal findings, they illustrate more broadly how individuals may modify movement-control strategies when sensory, cognitive, or functional demands are altered. These observations should therefore be interpreted as contextual rather than systematically synthesized evidence.

Taken together, the evidence included in the present review indicates that compensatory movement represents a context-dependent response that may simultaneously facilitate sport-specific performance and modify biomechanical loading [16,17,20]. Whether such adaptations remain beneficial or contribute to cumulative musculoskeletal stress is likely to depend on the interaction between technique, fatigue, functional capacity, sport-specific role, and exposure volume. The contextual literature [57,60,61] further supports the broader concept that movement strategies can change when motor-control demands are increased, but these studies should not be considered part of the systematic evidence base of this review. Within the evidence included in the review, repeated assessment of movement strategy under fatigue may help practitioners to identify adaptations that preserve sport-specific performance while potentially increasing localized mechanical loading.

### 4.6. Health Surveillance and Athlete Burden

The burden of injuries, illnesses and health issues in adaptive and Para sport athletes is substantial during training and competition seasons, as indicated in the longitudinal health-surveillance studies [22,32,39]. The research in this area widens the scope of adaptive sport away from just biomechanical or performance parameters. They demonstrate how repeated exposure, competition demands, past injury history, sex-related differences, impairment characteristics and variations in training intensity impact the health of athletes. Interestingly, morbidity also contributed significantly to the health burden, particularly respiratory and general medical conditions. This means that musculoskeletal injury is not the only health monitoring that should be applied to Para sport. Performance and training exposure must be interpreted in the context of the athlete’s overall health status (fatigue, sickness, recovery, and restriction to participate). Emerging evidence further indicates that lifestyle-related behaviors may contribute to the overall health burden experienced by adaptive populations. Excessive smartphone use has been associated with impaired postural balance among adolescents with intellectual disabilities, suggesting that non-sport factors may also influence physical function and should be considered within comprehensive athlete-monitoring systems [60,61]. From an applied perspective, athlete-health surveillance may therefore benefit from considering injury, illness, fatigue, recovery, and participation restriction together rather than focusing exclusively on musculoskeletal injury.

Based on the evidence gathered from this systematic review, it appears that adaptive and Para sport performance can be best understood as a dynamic balance between functional capacity, sport-specific skill, wheelchair configuration, biomechanical efficiency, workload exposure and health status. Taken together, the studies included in this review suggest that performance and health within the represented Para sport populations emerge from interactions among functional capacity, sport-specific skill, wheelchair or equipment configuration, biomechanical efficiency, workload exposure, and health status. In wheelchair tennis, these interactions are evident in trunk control, racket–wheelchair coordination, propulsion, and court mobility; in wheelchair basketball, they are reflected in mobility, functional classification, workload response, and upper-limb demands; and in wheelchair rugby, they are apparent in propulsion mechanics, fatigue-related adaptations, and wheelchair configuration. Para-cycling and mixed Paralympic cohorts additionally highlight the importance of longitudinal injury and illness surveillance. Overall, the evidence indicates that athletes employ task-specific movement and loading strategies in accordance with their functional characteristics and sport demands. However, the limited and heterogeneous evidence base does not permit universal conclusions across all Para sport disciplines. In practice, the available evidence supports an individualized monitoring approach in which functional characteristics, sport-specific performance, workload exposure, equipment demands, and health status are considered concurrently.

### 4.7. Contextual Evidence Beyond the Included Systematic Evidence Base

The following observations are presented solely as contextual literature and were not part of the 22 studies included in the systematic synthesis. Evidence from athletes with intellectual disabilities has reported associations between dynamic balance, agility, jumping ability, and sprint performance, while integrated balance, strength, and plyometric training has been associated with improvements in selected physical performance outcomes [55,57]. Additional studies involving individuals with intellectual disabilities have examined postural control under varying cognitive, visual, and sport-related conditions [58,59]. Research from pediatric and adolescent populations has also explored relationships among motor competence, movement behaviors, cognition, and postural balance, including potential effects associated with smartphone use [56,60,61]. Although these studies provide broader contextual perspectives on movement adaptation, balance, and motor control in populations with disabilities, they were not eligible for the present systematic review and were not used to derive the principal findings, evidence synthesis, practical implications, or conclusions of this review. These contextual studies should therefore be regarded as hypothesis-generating evidence that may guide future research rather than as a basis for direct practice recommendations within the sport populations synthesized in this review.

#### 4.7.1. Limitations of the Study

This review has several limitations. First, the review protocol was not prospectively registered, which reduces transparency regarding prespecified methodological decisions and should be considered when interpreting the review process. Second, although the eligibility framework encompassed adaptive and Para sport, the included evidence focused predominantly on wheelchair basketball, wheelchair rugby, and wheelchair tennis, with comparatively limited evidence from Para-cycling and broader Paralympic cohorts. The findings therefore should not be generalized to all Para sport disciplines or impairment groups. Third, the primary database strategy required multiple concept blocks simultaneously and may consequently have reduced search sensitivity, although simplified Google Scholar searches and citation-chasing procedures were used as supplementary retrieval approaches. Fourth, only full-text, English-language, peer-reviewed journal articles were included, creating the possibility of language and publication bias. Fifth, the included studies were heterogeneous in study design, sample characteristics, impairment profiles, outcome definitions, measurement instruments, and statistical approaches, and several studies involved small samples. These factors prevented meaningful quantitative pooling and limit causal interpretation. Finally, different design-specific risk-of-bias tools were required; although a common visual format was used for presentation, judgments from different instruments should not be interpreted as directly equivalent.

#### 4.7.2. Practical Implications of the Study

Given the limited and heterogeneous nature of the available evidence, the following implications should be regarded as provisional considerations rather than established best practice. Practitioners working in the wheelchair and Para sport contexts represented in this review may consider integrating sport-specific skill, wheelchair mobility, trunk and upper-body function, workload exposure, and athlete health within individualized assessment and training processes. Where appropriate, combining internal-load measures, external-load indicators, performance measures, and athlete-reported fatigue or symptoms may provide a more comprehensive representation of training demands than reliance on a single metric. Similarly, periodic assessment of shoulder symptoms, movement function, recovery, and illness may assist clinicians and coaching staff in identifying changes warranting further evaluation. However, prospective intervention studies are required before specific training, monitoring, or injury-prevention strategies can be considered evidence-based recommendations.

## 5. Conclusions

This systematic review finds that the interaction between impairment type, functional classification, trunk control, wheelchair mobility, upper-limb power, propulsion efficiency, and sport-specific workload are all important to consider when examining how adaptive and Para sport performance is developed. In the 22 studies included, athletes who had improved trunk function, explosive upper-body strength, sprint ability and wheelchair-handling efficiency tended to have improved performance outcomes. Concurrently, the biomechanics of wheelchair-based sports repeatedly load the upper limb and shoulder girdle and have potential for shoulder and upper-limb pain, overuse injury, asymmetry, movement adaptations due to fatigue, and interference with health. Injury and illness surveillance also suggests that shoulder issues, respiratory illness, variations in workload and previous injury history continue to be important issues in Para sport. So, improving performance is not independent of injury prevention, rehab, equipment optimization, and monitoring of health regularly. Coaches, clinicians and sport scientists are advised to use classification-based, individualized training programs with the aid of regular assessment of the internal load, external load, shoulder function, pain, fatigue and illness symptoms. Future studies should have larger numbers, longitudinal designs, consistent outcomes and sport-specific biomechanical assessments to further the evidence base. By combining training, workload monitoring, rehabilitation, wheelchair configuration and health surveillance, the adaptive and Para sport athlete can obtain a more holistic athlete-support system that can lead to safer and higher performance when managing training and workload.

## Figures and Tables

**Table 1:** Inclusion and exclusion criteria.

PICO Component	Inclusion Criteria	Exclusion Criteria
Population (P)	Studies involving adaptive sport or Para sport athletes, including wheelchair basketball, wheelchair rugby, wheelchair tennis, Para-cycling, and mixed Paralympic athlete populations. Participants could include athletes with spinal cord injury, cerebral palsy, amputation, spina bifida, musculoskeletal impairment, neurological impairment, visual impairment, impaired muscle power, or other eligible sport-related impairments.	Studies involving only non-disabled/able-bodied athletes without a clearly defined adaptive sport group. Studies involving general rehabilitation patients, sedentary individuals, general wheelchair users, or clinical populations not participating in organized adaptive sport were excluded.
Population level	Competitive, trained, elite, professional, collegiate, junior, national, or international adaptive sport athletes were eligible if they participated in structured sport training, competition, simulated sport tasks, field testing, or sport-specific performance assessment.	Studies involving recreational physical activity only, non-sport exercise programmes, general wheelchair mobility, or non-athlete clinical rehabilitation samples were excluded.
Intervention/Exposure/Condition (I)	Studies examining sport participation, training exposure, match play, simulated game tasks, sprint protocols, wheelchair propulsion, functional classification, trunk function, sport-specific training, injury-prevention programs, workload monitoring, biomechanical testing, wheelchair set-up testing, serve accuracy testing, upper-body strength testing, and competitive-season exposure were included. This includes interventions such as shoulder injury-prevention programs, discipline-specific training, intermittent sprint protocols, wheelchair mobility testing, and sport-specific performance testing.	Studies not involving sport-specific exposure, training, competition, wheelchair propulsion, biomechanical loading, functional classification, injury surveillance, adaptive sport performance testing, or wheelchair sport-specific physical assessment were excluded. Purely theoretical, qualitative-only, opinion-based, or narrative studies were excluded.
Comparison (C)	Eligible comparisons included pre–post comparisons, intervention versus control groups, training versus match/competition contexts, functional classification groups, trunk-function groups, impairment groups, sex groups, elite versus novice groups, junior versus adult/international groups, national versus international standard players, sport-type comparisons, injury versus non-injury status, and exposure-level comparisons. Single-group quantitative studies were also eligible if they reported measurable descriptive, correlational, predictive, or pre–post quantitative outcomes relevant to the review.	Studies without any quantitative comparison, grouping, baseline/post assessment, exposure contrast, correlation, prediction model, or extractable descriptive quantitative data were excluded. Studies reporting only qualitative perceptions without numerical outcomes were excluded.
Outcomes (O)	Studies had to report at least one quantitative outcome related to exercise exposure/adherence, biomechanical load, internal or external load, injury risk, musculoskeletal pain, health problems, compensatory movement patterns, wheelchair propulsion mechanics, wheelchair mobility performance, sport-specific skill performance, serve accuracy, trunk function, functional classification, range of motion, strength, upper-body explosive power, asymmetry, heart rate, RPE, player load, sprint performance, agility, injury/illness incidence, injury/illness prevalence, or injury burden.	Studies that did not report quantitative outcomes relevant to biomechanical load, injury risk, compensatory movement, sport exposure, sport-specific performance, wheelchair mobility, functional classification, or adaptive sport health surveillance were excluded. Studies without mean ± SD, incidence/prevalence, *p*-values, effect sizes, correlations, regression values, or other extractable numerical data were excluded.
Study design	Quantitative study designs were included: randomized or non-randomized experimental studies, pre–post intervention studies, cross-sectional studies, observational studies, prospective cohort studies, longitudinal surveillance studies, correlational field-test studies, predictive modeling studies, and biomechanical modeling/testing studies with quantitative data.	Reviews, systematic reviews, meta-analyses, bibliometric studies, editorials, commentaries, case reports, purely qualitative studies, conference abstracts without full data, and protocols without results were excluded.
Data requirement	Studies were included if they provided extractable quantitative data such as mean ± SD, group comparisons, pre–post values, *p*-values, t-values, effect sizes, correlations, regression values, incidence rates, prevalence values, hazard ratios, odds ratios, classification accuracy, or injury burden. This allowed preparation of study-characteristics tables and, where possible, effect-size or synthesis tables.	Studies were excluded if numerical data were insufficient for extraction or interpretation, or if outcomes were reported only narratively without statistical or descriptive values.
Sport context	Studies conducted in real sport, simulated sport, training, competition, match play, wheelchair ergometer, sprint testing, field testing, serve testing, agility testing, wheelchair mobility testing, or sport-specific laboratory settings were eligible if the task represented adaptive sport demands.	Studies conducted in non-sport laboratory tasks without relevance to adaptive sport movement, training, load, performance, or injury risk were excluded.
Publication type	Full-text peer-reviewed journal articles were included.	Non-peer-reviewed reports, theses, dissertations, unpublished data, magazine articles, blogs, and non-scientific sources were excluded.
Language	Full-text peer-reviewed journal articles published in English were eligible.	Articles for which an English-language full text was not available were excluded.
Timeframe	No publication-date restriction was applied. All studies meeting the eligibility criteria were eligible regardless of publication year.	No study was excluded solely on the basis of publication year.

**Table 2:** Characteristics of included quantitative studies on exercise exposure, biomechanical load, injury risk, and compensatory movement patterns in adaptive sport (n = 22).

Author(s), Year Country	Study Design	Sport/Population	Sample Characteristics	Exposure/Intervention/Condition	Comparison/Grouping	Main Outcomes/Variables	Measurement Tools/Instruments	Key Findings Relevant to Review
Bergamini et al., (2015) [15] Italy	This research employed a mixed-method approach including biomechanical characterization (using correlation and regression analysis) and a randomized controlled trial to evaluate a specific training intervention	Junior wheelchair basketball	12 athletes (10 males, 2 females) with a mean age of 17.1 ± 2.7 years and 4.5 ± 1.8 years of experience. The participants had various medical conditions, including paraplegia, myelomeningocele, poliomyelitis, spastic diplegia, below-knee amputation, and knee arthroprosthesis	12-week discipline- and population-specific training program was administered twice a week. The program focused on strength (using elastic bands for muscle groups like biceps and triceps) and coordination skills (such as spatial orientation, rhythm, and reaction)	Participants were randomly assigned to an experimental group (EG, N = 6), that received the specific training plus standard training, or a control group (CG, N = 6) that only underwent the standard training	Key performance indicators included the 20-m sprint test time (*t*20*mS*), push cycle duration and frequency, peak progression force (*Fp*), bilateral symmetry (*sym*), and intercycle variability (coefficient of variation for timing, force, and symmetry). Additionally, peak power output (*PO*) was measured as an indicator of aerobic physical capacity	Researchers used three wearable inertial measurement units (IMUs) (accelerometers) mounted on the wrists and the wheelchair backrest, a manual digital stopwatch, and an arm crank ergometer	PO, CVsym, CVΔt + functional classification predicted >80% variance in 20 m sprint time. Training improved EG propulsion mechanics: ↑ push frequency, ↑ progression force, ↑ symmetry, but no significant change in sprint time in either group. Likely reasons: manual timing error, short program, high baseline fitness. Biomechanical process measures captured changes missed by outcome time.
Cavedon et al., (2015) [12]; Italy	Cross-sectional observational study	Young wheelchair basketball players competing in the Italian Young Wheelchair Basketball Championship	N = 52 players (45 males, 7 females), mean age 18.1 ± 4.6 years, mean wheelchair basketball experience 6.1 ± 3.4 years; disabilities included spinal cord injury, cerebral palsy, spina bifida, etc.	Anthropometric measurements, body composition, and sport-specific field tests administered along with collection of game-related statistics. Players grouped into four functional ability classes (A–D) based on their classification points	Four functional ability classes (A-D): <br>—Class A: 0.5 points (Fascia Rossa) <br>—Class B: 1.0 and 1.5 points <br>—Class C: 2.0 and 2.5 points <br>—Class D: 3.0, 3.5 and 4.0 points	Anthropometry (sitting height, body circumferences, skinfolds), body composition (fat mass %), sport-specific field tests (5 m sprint, 20 m sprint with ball, suicide test, maximal pass, pass for accuracy, spot shot, lay-ups), game-related statistics (free-throw points, two- and three-point field goals, total points scored)	Harpenden anthropometer; Harpenden skinfold caliper; stopwatch; standardized sport-specific field tests designed for wheelchair basketball; official match score sheets	Sitting height positively correlates with performance outcomes and game-related statistics. Upper arm circumference, maximal pass, and lay-ups tests explain 42–59% of variance in scoring. Functional classification somewhat correlates with performance but shows large overlap among intermediate classes. Experience and skinfolds (body fat) do not influence performance. The study suggests that classification may require refinement to better represent functional performance. Training plans should focus on maximal pass and lay-ups skills.
De Witte et al., (2016) [13]; Netherlands	Observational, quantitative, comparative study using video analysis to quantify wheelchair-athlete activities during entire wheelchair basketball matches	Wheelchair basketball/male wheelchair basketball players	56 male wheelchair basketball players (27 national standard, 29 international standard). National standard: 8 guards, 12 forwards, 7 centers (mean classification 2.1–4.1). International standard: 10 guards, 12 forwards, 7 centers (mean classification 2.0–4.0	Observation of wheelchair-athlete activities during the entire wheelchair basketball matches	Playing standard: national standard vs. international standard players. Field position: guard vs. forward vs. center players	Absolute and relative duration and frequency of wheelchair movements and athlete control options. Specific activities analyzed: standing still, driving forward, driving backwards, rotating, braking, and blocking. Relationship between the player’s field position and classification	Video analysis of matches. Two high-definition video cameras (Casio EX-FH100). Systematic observation method based on clearly described and defined wheelchair-athlete activities, developed through interviews with coaches	Playing standard influenced mobility: national players showed ↑ in forward driving duration/frequency, while international players showed ↑ in rotations, rotation duration, and agility. National players showed ↓/shorter rotations. Field-position effect was minimal: guards, forwards, and centers showed similar wheelchair-handling patterns. Classification-position link: class 1–1.5 → guards; class 4–4.5 → centers.
Iturricastillo Urteaga et al., (2016) [36]; Spain	Observational, longitudinal study during a competitive season	Wheelchair basketball (WB)/highly trained male WB players	10 Spanish First Division male WB players (mean age 34 ± 8 years, 24 ± 12 years since injury, 11 ± 7 years WB training experience, 4–6 training h/wk). Players were classified according to the International Wheelchair Basketball Federation (IWBF), with classifications ranging from 1 to 4.5 (e.g., SCI, Spina bifida, Viral disease, Amputation, Knee injury)	Competitive wheelchair basketball match play over a 6-month season. Data from 16 matches were collected, totaling 111 individual observations	Comparison of objective heart rate (HR)-based match load (ML) methods (Edwards ML, Stagno TRIMPMOD) against subjective rating of perceived exertion (RPE)-based ML methods (respiratory sRPEres ML, muscular sRPEmus ML)	Edwards ML (AU), TRIMPMOD (AU), sRPEres ML (AU), sRPEmus ML (AU), Pearson product-moment correlation (r), coefficient of determination (R^2^) between HR- and RPE-based ML methods	HR telemetry using Polar Team Sport System; ML calculated by Edwards and TRIMPMOD methods. RPE-based ML assessed using 0–10 Foster RPE scale for respiratory and arm-muscle effort, multiplied by match duration	Edwards = 255.3 ± 66.3 AU; TRIMPMOD = 167.9 ± 67.1 AU; sRPEres = 521.9 ± 188.7 AU; sRPEmus = 536.9 ± 185.8 AU. RPE-based ML showed moderate correlations with Edwards ML (r = 0.629–0.648, *p* < 0.001) and TRIMPMOD (r = 0.627–0.668, *p* < 0.001), explaining only ~40% variance in HR-based ML. Individual responses varied, likely due to heterogeneous impairments. RPE-based ML is practical/cost-effective for global internal-load monitoring in WB, but should be used as a proxy, not a full substitute for HR-based ML.
Van Der Slikke et al., (2016) [49]; Netherlands	Field-based quantitative research conducted in regular training and tournament settings	Wheelchair basketball (male athletes)	30 elite male athletes: 13 national level (average age 24.9) and 17 international level (average age 26.0)	A 12 m sprint test was performed in the athletes’ own sports wheelchairs	Athletes were grouped by competition level (national vs. international) and classification (ranging from 1.0 to 4.5)	Sprint time, number of pushes, maximal isometric push force, and wheelchair kinematics (speed, acceleration, displacement)	Three Shimmer3 inertial sensors (two on hubs, one on frame), a Mecmesin AFG 1000 N force gauge for isometric force, and video analysis for push detection validation	International athletes were significantly faster (3.67 s vs. 4.10 s) and used fewer pushes (7.5 vs. 8.6) than national athletes. While classification (physical capacity) significantly correlated with sprint performance in the national group, this correlation was not present in the international group. This suggests that international athletes use superior technique or optimized wheelchair configurations (e.g., seat height) to mitigate physical limitations associated with lower classification.
García-Gómez & Pérez-Tejero, (2017) [28]; Spain	Cross-sectional descriptive study evaluating a sample of athletes via questionnaire	Wheelchair basketball (WB) players	51 players (21 females, 30 males) aged 15 to 45 years. Participants were members of the Spanish national team or sub-23 preselection and had used a manual wheelchair for at least one year. The most common disability was spinal cord injury	The study assessed the influence of shoulder pain (SP) on specific sport skills	Comparisons were made based on gender (male vs. female) and age groups	Perception of shoulder pain during specific sport skills (SS), including shooting, rebounding, one-handed long passes, and general WB situations (such as pushing, braking, and pivoting)	Shoulder Pain Index for Wheelchair Basketball (SPI-WB), a self-reported 40-item questionnaire adapted from the Wheelchair User’s Shoulder Pain Index (WUSPI)	Shoulder pain prevalence = 27.5%. Significant gender effect observed for shooting-related pain: females > males. No gender differences for rebounding, one-handed long passes, or general sport situations. Age showed no significant effect on shoulder-pain reporting. Females generally reported ↑ shoulder pain and were more likely to use a wheelchair for daily mobility.
Wilroy & Hibberd, (2018) [30]; USA	Pre and post-test intervention design	Collegiate wheelchair basketball athletes	Final sample of 7 participants (5 males, 2 females); ages 19–25 (mean 21.86); disabilities included paraplegia from spinal cord injury, spina bifida, cerebral palsy, and single-leg amputation; athletes had 5.8 ± 2.4 years of competition experience and were currently free of shoulder pain	A 6-week shoulder injury prevention program performed 3 times per week. The 5 min program included strengthening exercises with therapeutic bands (scapular retraction, external rotation, and shoulder flexion) and a partner internal rotation stretch	Pre-intervention vs. post-intervention assessment of the same group; no separate control group was used	Humeral internal and external rotation range of motion (ROM), and strength (humeral internal rotation, external rotation, and scapular retraction)	Digital inclinometer (baseline) for ROM; Lafayette handheld dynamometer for strength; and an injury history and participation questionnaire	Participants showed significant improvements in dominant-limb shoulder ROM: internal rotation increased by an average of 11.4° (*p* = 0.012) and external rotation increased by 8.0° (*p* = 0.032). There were no significant increases in shoulder or scapular muscle strength (*p* > 0.05).
Rietveld et al., (2019) [21]; Netherlands	Cross-sectional design	Wheelchair tennis players	21 elite players in total: 12 talented youth players (juniors) and 9 players at an international competition level (adults). Characteristics include a mix of impairments (e.g., amputation, spina bifida, spinal cord injury) and varying ITF rankings	Participants performed four wheelchair tennis field tests: a 20 m sprint, a Spider test for maneuverability, a Butterfly-sprint test, and the Illinois test. Each test was performed while players held their tennis racket	Comparison of performance between the talented junior group and the international adult group to assess construct validity	Inter-trial reliability (end times, ICCs, SEM) and construct validity (discriminative ability between groups). Specific mobility variables included linear/rotational velocity, linear/rotational acceleration, and push characteristics (e.g., number of pushes, cycle time)	Three inertial measurement units (IMUs) attached to the wheelchair frame and both wheels. Data were analyzed using MATLAB algorithms and SPSS version 22	All four tests demonstrated high inter-trial reliability for end times (ICCs 0.95–0.99). Construct validity was good, as international adults were significantly faster and achieved higher velocities and accelerations than juniors in most tests. However, the Illinois test showed a systematic learning effect between trials. The Spider, Butterfly-sprint, and Illinois tests were highly correlated, suggesting that they measure similar rotational components; the authors recommend using the 20 m sprint for sprinting and the Spider test for maneuverability.
Fagher et al., (2020) [39]; Sweden	52-week prospective cohort study	Swedish Paralympic athletes (candidates for or participants in previous Paralympic Games)	107 athletes (70 men, 37 women) with a median age of 29. Impairment types included physical (n = 79), visual (n = 22), and intellectual (n = 6). Participants included both wheelchair users (n = 53) and ambulatory athletes (n = 54)	Athletes self-reported sports-related injuries and illnesses (SRIIPS) every week for 52 weeks while engaging in their normal sports-specific training and competition	Subgroups were compared based on sex, age, type of sport (team vs. individual; summer vs. winter), impairment category, training load (low, middle, high), and history of severe injury/illness	Annual incidence proportions (IPs), incidence rates (IRs) per 1000 h of exposure, type of SRIIPS, severity (measured by time loss), and risk factors	Data were collected via an eHealth-based self-report application (using the Briteback^®^ survey tool). Diagnoses were categorized using ICD-10 codes. Training load was calculated as a training load rank index (TLRI)	Annual IP: injuries = 68%, illnesses = 77%. IR: injury = 6.9/1000 h; illness = 9.3/1000 h. Injury risk ↑ in team sports (HR 1.88), previous severe injury (HR 2.37), and males (HR 1.76). Common outcomes: infections = 84% of illnesses; the shoulder = most injured region (23%). Severity: 34% injuries are severe (≥21 days time loss). Impairment role: 59% reported impairment contributed to injury mechanism.
Franchin et al., (2020) [17]; Italy	A kinematic bidimensional analysis reporting preliminary results of an observational study on propulsion characteristics	Wheelchair rugby athletes from the Italian National Team	12 had a spinal cord injury (SCI), with 10 specifically having a cervical spinal cord injury (CSCI). One athlete had cerebral palsy. The group included players with various disability levels, with classification points ranging from 0.5 to 3.5	Athletes performed two sports-specific dynamic tests on an inertial drum bench using their personal rugby wheelchairs: sprinting test over 20 m. Wingate test: a sprint test with applied resistance exerted by the drum bench	Neuromuscular ability: comparison between “low-point” athletes (greater disability) and “high-point” athletes (greater motor function). Test type: comparisons between the sprint test and the Wingate test. Test progression: comparison of parameters at the start, middle, and end of each test	Kinematic parameters: contact angle, release angle, total push angle, absolute arm angle, arm-to-forearm angle, and elbow flexion angle. Propulsion patterns: identification of trajectories (e.g., semi-circular, arcing, or double-loop-over). Range of motion (ROM): specifically, elbow ROM during different stages of propulsion. Temporal parameters: percentage of time spent in the push phase	Inertial drum bench. BTS Bioengineering Smart DX-6000 Optoelectronics Motion Capture 3D system with 8 infrared cameras. Canon PowerShot SX240 HS camera. Kinovea software (version 0.8.25.0) for video analysis of propulsion cycles	Propulsion style: most athletes, especially ↑ disability/low-point players, used a semi-circular pattern → protective for upper limbs; high-point players used an arching pattern → ↑ efficiency but ↑ joint stress. Speed ↑ strategy: ↑ release angle + ↓ contact angle → forward push angle, but ↑ shoulder load. Disability-specific kinematics: C5-C6/low-point players showed ↓ peak extension + ↑ elbow flexion to maximize biceps due to limited triceps use. Test demand: Wingate required ↑ more pushing time than the sprint test (50.1% vs. 44.4%). Key implication: a performance-efficient sprint technique may conflict with shoulder-pain prevention.
Ju et al., (2021) [20]; Taiwan	Comparative cross-sectional study	Wheelchair (WC) tennis players and able-bodied (AB) tennis players	30 total participants: 15 WC players (14 male, 1 female; mean age 41.2) and 15 AB collegiate varsity players (15 male; mean age 28.6). WC impairments included poliomyelitis (11), spinal cord injury (3), and bilateral amputation (1)	Participants performed single-handed backhand strokes against balls served by a pitching machine at 80 kph	Three conditions were compared: wheelchair players (sitting), able-bodied players in sitting positions, and able-bodied players in standing positions	Backhand swing time; trunk and shoulder kinematics (angular position/excursion, maximum velocity, and acceleration); and vibration intensity at the racket, wrist, and elbow	Liberty 3D electromagnetic motion analysis system (120 Hz); wearable tri-axial accelerometers (Crossbow); and a JUGS Combo Pitching Machine	Relevant to review: WC players demonstrated significantly larger forward trunk rotation and higher trunk/shoulder angular velocity and acceleration than standing AB players to compensate for a deficient kinetic chain. Movement patterns for trunk flexion/extension were reversed in sitting vs. standing positions. WC players used large, rapid shoulder abduction/adduction, not typical in AB players, which may increase injury risk. Finally, while racket vibration was lower in the WC condition, the vibration absorption (reduction ratio) from racket to wrist was significantly less efficient in WC players.
Demeco et al., (2022) [29]; Italy	Pilot study (longitudinal intervention)	Wheelchair basketball athletes	10 male professional athletes (mean age 33.75 ± 6.42 years) playing in the Italian Second League. Diagnoses included lower limb amputations (n = 4), spinal cord injuries (n = 2), spina bifida (n = 1), cerebral palsy (n = 1), rickets (n = 1), and osteogenesis imperfecta (n = 1)	An 8-week comprehensive rehabilitative program (4 sessions/week) consisting of: education on injury avoidance, ergonomics training (modifying task performance), flexibility and stretching, and strengthening exercises using elastic bands focused on shoulder depressors and scapular stabilizers	Pre-intervention (T0) vs. post-intervention (T1)	Shoulder pain during functional activities, functional status of upper extremities, shoulder range of motion (ROM) (flexion, abduction, extension, internal/external rotation), muscle activity (sEMG), and propulsion patterns (acceleration, symmetry, and smoothness)	Wheelchair User’s Shoulder Pain Index (WUSPI); Kerlan-Jobe Orthopaedic Clinic (KJOC) questionnaire; kinematic analysis via an optoelectronic system with infrared cameras; surface electromyography (sEMG); and an inertial measurement unit on a training roller	Significant reduction in shoulder pain (WUSPI) and improvement in functional scores (KJOC). There was a significant increase in scapular upward rotation, abduction, and external rotation of the glenohumeral joint. Muscle activation patterns shifted with decreased deltoid contribution and increased activity in the teres minor and serratus anterior, aiding joint stability. Propulsion became more efficient, fluid, and symmetrical.
Fapojuwo et al., (2022) [27]; Nigeria	Cross-sectional survey	Wheelchair athletes participating in local and international competitions. Specific sports included wheelchair basketball, racing, tennis, volleyball, and karate	The study included 63 wheelchair athletes. The mean age was 30.79 ± 7.29 years (range 16–48). Participants were 58.7% male and 41.3% female. The primary cause of wheelchair use was poliomyelitis (69.8%), followed by amputation (23.8%), trauma (3.2%), and spinal cord injury (3.2%). Most (71.4%) had participated in sports for 1–10 years	The condition studied was the occurrence of musculoskeletal injuries related to wheelchair sports training or competition	The study analyzed associations between 12-month injury prevalence and variables including age, sex, duration of sports participation, type of sport, and specific injured body parts	Key variables measured were injury prevalence (career, 12-month, and point), injury pattern (site and nature), perceived risk factors, injury severity, and prevention/treatment strategies	An adapted questionnaire was used, based on the “daily report on injuries and illnesses, code and classification” and the “injury and illness definitions and data collection procedures for epidemiological studies in athletics”	Injury prevalence was high: career = 93.7%, 12-month = 61.9%. Common sites: shoulder (66.7%) > forearm (31.7%). Common types: cramps/spasms (57.1%), strains (20.6%), bruises (20.6%). Main perceived risks: fatigue (30.2%) + athlete contact (28.6%). Most injuries occurred during training (81%). Many continued training injured (79.4%) and used self-medication (54%). Protective gear non-use was high (61.9%), mainly due to perceived non-necessity/financial limits. No significant association with age, sex, or years of participation.
Haydon et al., (2022) [50]; Australia	Original research utilizing a two-step modeling approach: first, adapting a linkage model to predict propulsion kinematics, and second, applying partial least-squares (PLS) regression to predict performance. The testing phase used an orthogonal design (L9 orthogonal array) to systematically vary wheelchair parameters	Elite wheelchair rugby (WCR) players	Eight elite male players from the Australian WCR team. Participants had various impairments, including limb deficiency and impaired muscle power, with classification scores ranging from 1.0 to 3.5 points	Participants completed 5 m sprints from a standstill in nine different wheelchair set-ups. Four parameters were varied across three levels (current, increased, and decreased): seat height (±15 mm); seat depth (±15 mm); seat angle (±5 degrees); tire pressure (±15 psi)	The study compared experimental (on-court) data against predicted data generated by the models. The PLS regression was trained using data from seven set-ups and then tested on the remaining two set-ups to evaluate prediction accuracy	Propulsion kinematics: hand contact and release angles for the first three strokes. Performance: 5 m sprint time. Anatomical variables: trunk angles at contact and release	Adjustable wheelchair (14 kg) with 25-inch wheels. Laser timing gates (SpeedLight, Swift Performance) for sprint times. Digital video (120 Hz, GoPro Hero 3+) for sagittal plane kinematic analysis. Inertial measurement units (IMUs) (500 Hz, IMeasureU) used to identify contact and release moments via acceleration spikes. Matlab (Mathworks) for custom analysis scripts and PLS regression	Model accurately predicted contact angles: error < 5° in 43/48 predictions; 75% within 0.5° of experimental values. Release-angle prediction was weaker, with ↑ errors in 2nd–3rd strokes. Sprint-time prediction varied individually (error: 0.01–0.87 s). Athlete-specific limitations/propulsion style affected accuracy, suggesting need for individualized/musculoskeletal models. Approach may ↓ costly/time-consuming on-court wheelchair-optimization testing.
Alim et al., (2023) [18]; Indonesia	Quantitative study utilizing correlation analysis conducted through field tests	Wheelchair tennis (WT) players	The study involved 10 professional wheelchair tennis athletes (7 men and 3 women) from the National Paralympic Committee of Yogyakarta Special Region. The participants had a mean height of 167 ± 5.6 cm, weight of 59.60 ± 6.59 kg, and age of 33.4 ± 1.51 years. Their disabilities included polio (n = 6), amputation (n = 1), spinal cord injury (n = 2), and impaired muscle power (n = 1), with nine athletes in the “open” category and one in the “quad” category	Participants underwent a series of neuromuscular and physical field tests over two consecutive days. These tests included: isometric handgrip strength; serve accuracy (Hewitt test); sprint tests at 5 m, 10 m, and 20 m, both with and without rackets; agility (T-test) with and without rackets; medicine ball throws (2 kg) simulating serve, forehand, and backhand movements; anaerobic endurance (Hit and Turn Tennis Test)	The study primarily analyzed the correlation between various physical parameters (independent variables) and service accuracy (dependent variable). For logistic regression analysis, athletes were categorized into two groups based on their serve accuracy: (1) good and excellent, and (2) average, poor, and very poor	The primary outcome was serving accuracy. The main independent variables were physical factors, including explosive upper-body strength (medicine ball throws), sprint speed, agility, grip strength, and anaerobic endurance	Hewitt service test: used to measure serve placement accuracy. Hand dynamometer: measured maximal isometric finger flexor strength. Stopwatch and field markers: used for sprint (5 m, 10 m, 20 m) and T-test agility measurements. 2 kg medicine ball: used to measure explosive strength via simulated hitting movements. Acoustically controlled signal: used for the Hit and Turn Tennis Test to assess anaerobic endurance. IBM SPSS 25.00: used for statistical analyses including Shapiro–Wilk (normality), Pearson correlation, and binary logistics regression	MBT-serve showed the strongest + correlation with SA (r = 0.874, *p* = 0.001), followed by MBT-forehand (r = 0.811, *p* = 0.004) and MBT-backhand (r = 0.747, *p* = 0.013). Sprint 20 m with racket showed a significant − correlation with SA (r = −0.718, *p* = 0.019), indicating faster sprint time → higher SA. MBT variables strongly predicted SA level (Nagelkerke R^2^ = 1.00; classification accuracy = 100%), but this should be interpreted cautiously due to small n. Grip strength, agility, and endurance showed no significant association with SA (*p* > 0.05). Overall, trunk rotational power and upper-body explosive strength appear important for SA in wheelchair tennis.
Briley et al., (2023) [16]; Netherlands	This was an original experimental study utilizing a two-way mixed analysis of variance (ANOVA) to examine the effects of time (pre vs. post) and impairment (SCI vs. non-SCI)	Elite international wheelchair rugby (WR) players	The sample included 15 male international players with a mean age of 30.3 ± 5.5 years. Participants were divided into two groups: those with spinal cord injury (SCI; n = 7, levels C5-C7) and those with non-SCI health conditions (n = 8), which included amputations, cerebral palsy, Roberts syndrome, and others	Participants completed a sports-specific intermittent sprint protocol (ISP) designed to simulate the physiological demands of a wheelchair rugby match. The ISP lasted 73 min and consisted of four 16 min quarters with varying propulsion speeds based on the player’s peak velocity	The study compared biomechanical and physiological data before (pre-ISP) and immediately after (post-ISP) the protocol. It also compared outcomes between the SCI and non-SCI impairment groups	Sprint performance: peak velocity (*Vmax*) and total distance traveled during 10 s sprints. Physiological parameters: heart rate (HR), blood lactate concentration (BLa), and rating of perceived exertion (RPE). Biomechanical variables: thorax flexion, glenohumeral (GH) flexion and abduction (peak angles and range of motion), contact angles, peak force, peak power, and inter-limb asymmetries for these parameters	Dual roller wheelchair ergometer (Lode Esseda) for kinetic and spatio-temporal data. 10-camera Vicon motion analysis system (200 Hz) for upper-body kinematics. Physiological tools: Polar Team2 for heart rate, Biosen C-line for blood lactate, and Borg’s 6–20 scale for RPE	Performance maintained: despite ↑ BLa, HR, and RPE, sprint peak velocity and distance remained unchanged post-ISP. Biomechanical adaptation: post-ISP showed ↓ thorax flexion and ↓ peak GH abduction during acceleration/max-velocity phases. Asymmetry ↑: ↑ contact-angle and GH-flexion asymmetry during acceleration; ↑ GH-abduction asymmetry at max velocity. SCI-specific effect: SCI players showed ↑ peak-power asymmetry (+6%) and ↑ GH-abduction asymmetry (+15%) post-ISP; non-SCI did not. Protective strategy: ↓ GH abduction may ↑ increase the risk of subacromial space and ↓ acute shoulder pain/fatigue risk.
Esak et al., (2024) [19]; Malaysia	Experimental correlation study (preceded by a pilot study with 12 non-disabled participants)	Wheelchair tennis athletes	9 national-level wheelchair tennis athletes (5 elite and 4 novice); 7 males and 2 females; 6 with spinal cord injury (SCI) and 3 with amputation/limb differences (ALDs); weights range from 54 to 70 kg; athletic experience ranges from 2 months to 6 years	Three propulsion activities performed on a standard hardcourt: a 10 m comfortable speed test, a 10 m sprint test, and a 20 m round-trip sprint test	Comparisons were conducted between elite vs. novice players, propelling with racket vs. without racket, and between injury types (SCI vs. ALD)	Push frequency (number of pushes per second), stroke time (duration of one complete push cycle), average propulsion velocity (m/s), and rate of perceived exertion (RPE)	Nikon D5600 DSLR camera for high-resolution video recording, Kinovea software for data extraction and velocity analysis, measuring tape for calibration, and the Borg scale (0–10) for RPE assessment	PF ↑ was strongly associated with PV ↑ (r = 0.840, *p* < 0.001), while ST ↑ was strongly associated with PV ↓ (r = −0.859, *p* < 0.001). Elite athletes showed higher PF and PV than novice athletes. Racket holding ↓ maximal speed and ↑ RPE compared with propulsion without racket. RPE showed positive associations with PV and PF, but a negative association with ST. Athletes with ALD showed a trend toward higher PF and PV than athletes with SCI, although this was not statistically tested due to small sample size.
Hernández-Beltrán et al., (2023) [51]; Spain & Portugal	Cross-sectional design with an associative and descriptive strategy	Wheelchair basketball/national wheelchair basketball team players (n = 12)	12 players from a national WB team (age = 31.5 ± 8.3 years, height = 167.0 ± 0.25 cm, weight = 68.0 ± 12.6 kg), with functional classifications (FCs) ranging from 1.0 to 4.5	Training sessions and two official matches, focusing on 5 × 5 game situations	Sporting context (training vs. competition); functional classification (FC 1.0 to 4.5)	External load (EL) and internal load (IL)	WIMU PRO™ inertial devices (for EL) and Garmin™ HR bands (for IL)	Significant differences were found across contexts, with higher external- and internal-load values during competition than during training sessions. Players with a higher functional classification generally exhibited more differences and higher load values. Understanding these loads is crucial for personalizing training and reducing injury risk.
Hernández-Beltrán et al., (2024) [14]; Spain, Chile, Portugal	Observational, cross-sectional study using simulated 5 × 5 wheelchair basketball game tasks under controlled conditions	Wheelchair basketball (WB); male national professional WB players	12 male national professional WB players; age 30.7 ± 4.82 years; WB experience 5 ± 1.43 years; grouped by functional classification (FC) from 1.0 to 4.0	Participation in 5 × 5 wheelchair basketball simulated games designed to reproduce match physical demands; monitoring external and internal load data during play sessions	Grouped by functional classification (FC): players with different mobility/trunk control scores (1.0 to 4.0); comparisons of load variables between FC groups	External-load (EL) kinematic variables: distance, explosive distance, acceleration (acc), deceleration (dec), max. acceleration, max. deceleration, average speed, max. speed. External-load neuromuscular variables: player load (PL), impacts. Internal-load (IL) variables: average heart rate (HR), max HR, % of max HR	- WIMU PRO™ inertial devices with ultra-wide band (UWB) system for external-load data (RealTrack Systems, Spain). Garmin™ heart rate monitor bands for internal-load measurements	Higher FC (4.0) showed ↑ EL kinematics (distance, deceleration, avg speed), ↑ neuromuscular load (PL, impacts), and ↑ IL (HR). FC2.0 showed the highest explosive distance, reflecting intermittent/explosive demands. Greater functional capacity → ↑ mobility/trunk control → ↑ physical + cardiovascular load. Anthropometrics/experience related to max acceleration/speed. Findings support FC-based individualized load prescription to ↑ performance and ↓ injury risk.
Busch et al., (2025) [22]; Germany	Prospective cohort study conducted over an observation period of 124 weeks	Elite Paralympic athletes who were members of the German Paralympic squad	The study analyzed 122 participants (58 females and 64 males) with a mean age of 28 years (range 16–61). The cohort included athletes with various impairments: musculoskeletal (33.6%), impaired range of motion (34.4%), neurological (18%), visual (12.3%), and intellectual (1.6%). Approximately 50.8% were ambulatory and 49.2% used wheelchairs for sports	Monitoring of health problems (injuries and illnesses) in relation to sporting exposure, including regular training, training camps, competitions, and recovery periods	Comparisons were made based on sex (female vs. male), mode of mobility (ambulatory vs. wheelchair), impairment types, years of elite training (<5 years, 5–10 years, and >10 years), and primary weekly sporting activity	The primary outcomes were the prevalence, incidence, and burden (time-loss days) of health problems. Secondary variables included training exposure hours and subjective training intensity	Oslo Sports Trauma Research Centre questionnaire on health problems (OSTRC-HP), administered weekly via an online application. Sports Medicine Diagnostic Coding System (SMDCS) for classifying diagnoses; four-point Likert scale for recording subjective training intensity	Health-problem prevalence: 16.6% at any time; substantial health problems = 10.5%. Risk ↑: females had ~2× higher risk than males (HR = 1.8). Experience protective: >5 yrs elite training ↓ risk; OR = 0.9/year. Seasonality: overuse injuries peaked in training camps (41.2%); acute injuries highest in competitions (31.8%). Training intensity changes above/below normal ↑ odds of substantial health problems. Most burdensome: shoulder injuries + respiratory infections.
Fallon et al., (2025) [32]; Northern Ireland and Ireland	A single-center, one-season prospective longitudinal study	Professional endurance Para-cycling	Ten professional Paracyclists (5 male, 5 female) are preparing for the 2024 Paris Paralympic Games. Impairments included spinal cord-related, neuromuscular, and musculoskeletal conditions, as well as vision impairment	A 35-week monitoring period during the 2024 Paralympic season (February to October). Exposure included 2430 training days and 410 competition days	Data was compared between sexes (male vs. female) and between different participation statuses (e.g., full participation vs. health problem)	Incidence and rates of injury and illness (expressed per 365 days and 1000 h), injury/illness severity and burden, and subjective well-being markers (fatigue, stress, mood, and sleep quality)	The Oslo Sports Trauma Research Centre Questionnaire on Health Problems V2 (OSTRC-H2), medical logs, and Training Peaks software (Version no. V12.74.0) for tracking training metrics (time, distance, energy expenditure, and training stress score)	Health problems: 7.36/365 days; medical attention = 5.56/365 days. Injury rate: 1.94/365 athlete-days; males > females (2.44 vs. 1.51). Illness rate: 3.60/365 athlete-days; females > males (5.40 vs. 1.80). Shoulder = the most common injury site. Respiratory illness = most common illness (56%); females >3× more likely than males. Health-problem reporting was linked with ↑ perceived fatigue (*p* < 0.001).
Van Der Slikke et al., (2026) [10]; Netherlands	Cross-sectional observational study	Wheelchair tennis (WT); elite international players	51 players total: 24 men, 12 women, and 15 quad (all male). All were elite-level athletes participating in major 2023 international tournaments	Standardized on-court field tests (e.g., 20 m and 12 m sprints, slalom, 180° turns) performed with and without a racket, and match play analysis	Athletes were grouped by trunk function scores (0: non-functional; 1: partial control; 2: full control). Exploratory comparisons were also made between dominant/non-dominant arms and mobility with/without a racket	Strength metrics: isometric push/pull forces on the rim and isolated arm forces (flexion/extension). Mobility metrics: maximal speed, forward and rotational acceleration, maximal/average rotational speed, trunk angle, and backward trunk acceleration	Mecmesin Advanced Force Gauge with a 1000 N S-Beam load cell for strength. Inertial measurement units (IMUs) (xIMU3) were used to capture wheelchair and trunk kinematics	Trunk function significantly impacts mobility, particularly in rotation and acceleration, with large effect sizes (ES = 1.18–2.43) between full and minimal trunk control. Upper-body strength shows only modest correlations (r = 0.26–0.62) with mobility performance. Racket use reduces maximal speed and increases trunk angle. The study concludes that trunk function is underrepresented in the current classification system (accounting for only 14% of the score) and should be weighted more heavily based on its functional impact.

Note: wheelchair basketball (WB); wheelchair rugby (WR); Para-cycling (PC); and broader Paralympic athlete populations; with sample sizes ranging from small pilot studies to large prospective cohorts. Participants varied in age; sex; impairment type; functional classification (FC); cross-sectional (CS); experimental (EXP); pilot intervention (PI); observational (OBS); and prospective cohort (PCoh) approaches; external load (EL); internal load (IL); propulsion biomechanics (PB); shoulder pain (SP); injury prevalence (IP); functional classification (FC); and sport-specific performance (SSP). Measurement tools commonly included inertial measurement units (IMUs); heart-rate (HR) monitors; motion analysis systems (MASs); questionnaires; and clinical assessment tools (CATs).

**Table 3:** Risk-of-bias assessment of included studies.

No.	Study	Tool	Selection/Sampling	Confounding	Exposure/Intervention/Performance Measurement	Outcome Measurement	Missing Data	Statistical Analysis	Overall
1	Bergamini et al. (2015) [15]	RoB 2	🟡	🟡	🟢	🟡	🟡	🟡	🟡
2	Cavedon et al. (2015) [12]	JBI Cross-sectional	🟡	🟡	🟢	🟡	🟢	🟡	🟡
3	De Witte et al. (2016) [13]	JBI Cross-sectional	🟡	🟡	🟢	🟡	🟢	🟡	🟡
4	Iturricastillo Urteaga et al., (2016) [36]	JBI Cross-sectional	🟡	🟡	🟢	🟢	🟡	🟡	🟡
5	Van der Slikke et al. (2016) [49]	JBI Cross-sectional	🟡	🟡	🟢	🟡	🟢	🟡	🟡
6	García-Gómez & Pérez-Tejero, (2017) [28]	JBI Cross-sectional	🟡	🟡	🟡	🟡	🟢	🟡	🟡
7	Wilroy & Hibberd (2018) [30]	ROBINS-I	🟡	🔴	🟢	🟢	🟡	🟡	🔴
8	Rietveld et al. (2019) [21]	JBI Cross-sectional	🟡	🟡	🟢	🟢	🟡	🟢	🟡
9	Fagher et al., (2020) [39]	JBI Cohort	🟡	🟡	🟢	🟡	🟡	🟢	🟡
10	Franchin et al., (2020) [17]	JBI Cross-sectional	🟡	🟡	🟡	🟡	🟢	🟡	🟡
11	Ju et al. (2021) [20]	JBI Cross-sectional	🟡	🟡	🟢	🟢	🟢	🟡	🟡
12	Demeco et al., (2022) [29]	ROBINS-I	🟡	🔴	🟢	🟡	🟡	🟡	🔴
13	Fapojuwo et al. (2022) [27]	JBI Cross-sectional	🟡	🔴	🟡	🟡	🟡	🟡	🔴
14	Haydon et al., (2022) [50]	JBI Cross-sectional	🟡	🟡	🟢	🟡	🟢	🟡	🟡
15	Alim et al. (2023) [18]	JBI Cross-sectional	🟡	🔴	🟡	🟡	🟢	🔴	🔴
16	Briley et al., (2023) [16];	JBI Cross-sectional/repeated-measures	🟡	🟡	🟢	🟢	🟢	🟡	🟡
17	Esak et al. (2024) [19]	JBI Cross-sectional	🟡	🟡	🟡	🟡	🟢	🟡	🟡
18	Hernández-Beltrán et al. (2023) [51]	JBI Cross-sectional	🟡	🟡	🟢	🟢	🟢	🟡	🟡
19	Hernández-Beltrán et al. (2024) [14]	JBI Cross-sectional	🟡	🟡	🟢	🟢	🟢	🟡	🟡
20	Busch et al. (2025) [22]	JBI Cohort	🟡	🟡	🟢	🟡	🟡	🟢	🟡
21	Fallon et al., (2025) [32]	JBI Cohort	🟡	🟡	🟢	🟡	🟡	🟡	🟡
22	Van der Slikke et al. (2026) [10]	JBI Cross-sectional	🟡	🟡	🟢	🟢	🟢	🟢	🟡

Note: RoB 2 = Cochrane Risk of Bias 2 tool; ROBINS-I = Risk Of Bias In Non-randomized Studies of Interventions; JBI = Joanna Briggs Institute; JBI Cross-sectional = Joanna Briggs Institute Critical Appraisal Checklist for Analytical Cross-Sectional Studies; JBI Cohort = Joanna Briggs Institute Critical Appraisal Checklist for Cohort Studies; ROM = range of motion; IMU = inertial measurement unit; HR = heart rate; RPE = rating of perceived exertion; sEMG = surface electromyography; 🟢 = low risk of bias/adequate methodological reporting; 🟡 = some concerns/unclear methodological reporting; 🔴 = serious or high risk of bias.

## Data Availability

No new data were generated or analyzed during this study. All relevant information and original contributions are fully presented within the article. Therefore, data sharing is not applicable to this manuscript.

## Supplementary Materials

The following supporting information can be downloaded at: https://www.mdpi.com/article/10.3390/sports14090396/s1, Table S1. Quantitative Summary of Included Adaptive and Para Sport Studies (n = 22).
