# Supplementary material for: Performance, Biomechanical Demands, Injury Burden, and Health Surveillance in Wheelchair Court Sports and Selected Para Sport Cohorts: A Systematic Review
Source: Sports (Basel). 2026 Sep 7;14(9):396. doi: 10.3390/sports14090396 (PMC13611318; doi:10.3390/sports14090396)
Supplement: Supplementary file 1 [file sports-14-00396-s001.zip › PRISMA_2020_checklist PARA26.pdf]

PRISMA

PRISMA 2020 Checklist

Section and Topic	Item #	Checklist item	Location where item is reported
**TITLE**			
Title	1	Identify the report as a systematic review.	Page 1, lines 1–3
**ABSTRACT**			
Abstract	2	See the PRISMA 2020 for Abstracts checklist.	Page 2, lines 36–71 , page 3, lines 72–75
**INTRODUCTION**			
Rationale	3	Describe the rationale for the review in the context of existing knowledge.	Pages 3–5, lines 78–166
Objectives	4	Provide an explicit statement of the objective(s) or question(s) the review addresses.	Page 5, lines 167–175
**METHODS**			
Eligibility criteria	5	Specify the inclusion and exclusion criteria for the review and how studies were grouped for the syntheses.	Pages 5–6, lines 177–209; page 9, line 327 - page 11
Information sources	6	Specify all databases, registers, websites, organisations, reference lists and other sources searched or consulted to identify studies. Specify the date when each source was last searched or consulted.	Page 6, lines 210–222; page 7, lines 266–278.
Search strategy	7	Present the full search strategies for all databases, registers and websites, including any filters and limits used.	page 6, lines 223–236; page 7, lines 237–251; page 7, lines 253–265; page 7, lines 266–274
Selection process	8	Specify the methods used to decide whether a study met the inclusion criteria of the review, including how many reviewers screened each record and each report retrieved, whether they worked independently, and if applicable, details of automation tools used in the process.	page 8, lines 279–309 and page 9, lines 310–317
Data collection process	9	Specify the methods used to collect data from reports, including how many reviewers collected data from each report, whether they worked independently, any processes for obtaining or confirming data from study investigators, and if applicable, details of automation tools used in the process.	Page 11, lines 339–358.
Data items	10a	List and define all outcomes for which data were sought. Specify whether all results that were compatible with each outcome domain in each study were sought (e.g. for all measures, time points, analyses), and if not, the methods used to decide which results to collect.	Page 6, lines 196–202; page 11, lines 349–356; page 32, lines 453–459
	10b	List and define all other variables for which data were sought (e.g. participant and intervention characteristics, funding sources). Describe any assumptions made about any missing or unclear information.	Page 11, lines 344–349; Table 1, pages 9–11.
Study risk of bias assessment	11	Specify the methods used to assess risk of bias in the included studies, including details of the tool(s) used, how many reviewers assessed each study and whether they worked independently, and if applicable, details of automation tools used in the process.	page 12, lines 360–393; page

PRISMA

PRISMA 2020 Checklist

Section and Topic	Item #	Checklist item	Location where item is reported
			32, lines 465–471, page 34, lines 472–488
Effect measures	12	Specify for each outcome the effect measure(s) (e.g. risk ratio, mean difference) used in the synthesis or presentation of results.	Page 11, lines 352–356; page 13, lines 405–414
Synthesis methods	13a	Describe the processes used to decide which studies were eligible for each synthesis (e.g. tabulating the study intervention characteristics and comparing against the planned groups for each synthesis (item #5)).	Page 12, lines 394–398; page 13, lines 399–405
	13b	Describe any methods required to prepare the data for presentation or synthesis, such as handling of missing summary statistics, or data conversions.	Page 11, lines 352–358; page 13, lines 407–410
	13c	Describe any methods used to tabulate or visually display results of individual studies and syntheses.	page 13, lines 430–439; page 14, lines 440–442; page 31; page 32, line 471
	13d	Describe any methods used to synthesize results and provide a rationale for the choice(s). If meta-analysis was performed, describe the model(s), method(s) to identify the presence and extent of statistical heterogeneity, and software package(s) used.	Page 12, lines 394–398; page 13, lines 399–414; page 38, lines 667–668
	13e	Describe any methods used to explore possible causes of heterogeneity among study results (e.g. subgroup analysis, meta-regression).	Page 12, lines 395–398; page 13, lines 399–405
	13f	Describe any sensitivity analyses conducted to assess robustness of the synthesized results.	Not reported
Reporting bias assessment	14	Describe any methods used to assess risk of bias due to missing results in a synthesis (arising from reporting biases).	Not reported
Certainty assessment	15	Describe any methods used to assess certainty (or confidence) in the body of evidence for an outcome.	Not reported
**RESULTS**			
Study selection	16a	Describe the results of the search and selection process, from the number of records identified in the search to the number of studies included in the review, ideally using a flow diagram.	page 8, lines 279–297; page 9, lines 310–317; page 13, lines 416–429
	16b	Cite studies that might appear to meet the inclusion criteria, but which were excluded, and explain why they were excluded.	page 8, lines 279–297
Study characteristics	17	Cite each included study and present its characteristics.	Page 13, lines 430–439; Table 2, page 14, lines 440–442 to page

Section and Topic	Item #	Checklist item	Location where item is reported
			31; page 31, lines 443–446 page 32, lines 447–464
Risk of bias in studies	18	Present assessments of risk of bias for each included study.	Table 3: page 32, lines 465–471; table continues page 33; explanatory note and interpretation: page 34, lines 472–488
Results of individual studies	19	For all outcomes, present, for each study: (a) summary statistics for each group (where appropriate) and (b) an effect estimate and its precision (e.g. confidence/credible interval), ideally using structured tables or plots.	Table 2: pages 14–31, beginning page 14, lines 440–442; supplementary quantitative details: Supplementary Table S1, pages 1–13
Results of syntheses	20a	For each synthesis, briefly summarise the characteristics and risk of bias among contributing studies.	page 13, lines 416–439; risk-of-bias summary: page 32, lines 465–470 and page 34, lines 480–488
	20b	Present results of all statistical syntheses conducted. If meta-analysis was done, present for each the summary estimate and its precision (e.g. confidence/credible interval) and measures of statistical heterogeneity. If comparing groups, describe the direction of the effect.	No meta-analysis/statistical synthesis performed. Narrative synthesis/results: page 13, lines 416–439; Table 2 pages 14–31; supplementary quantitative findings in Supplementary Table S1, pages 1–13; no meta-analysis stated page 38, lines

Section and Topic	Item #	Checklist item	Location where item is reported
			667–668
	20c	Present results of all investigations of possible causes of heterogeneity among study results.	Methods: page 12, lines 395–398 and page 13, lines 399–405; limitations: page 38, lines 650–668
	20d	Present results of all sensitivity analyses conducted to assess the robustness of the synthesized results.	Not reported
Reporting biases	21	Present assessments of risk of bias due to missing results (arising from reporting biases) for each synthesis assessed.	**Not reported.**
Certainty of evidence	22	Present assessments of certainty (or confidence) in the body of evidence for each outcome assessed.	Not reported.
**DISCUSSION**			
Discussion	23a	Provide a general interpretation of the results in the context of other evidence.	page 34, lines 491–507; thematic discussion: page 34, line 508 to page 38, line 648
	23b	Discuss any limitations of the evidence included in the review.	Page 38, lines 649–668
	23c	Discuss any limitations of the review processes used.	Page 38, lines 664–668,
	23d	Discuss implications of the results for practice, policy, and future research.	page 38, lines 669–683; page 39, lines 684–704
**OTHER INFORMATION**			
Registration and protocol	24a	Provide registration information for the review, including register name and registration number, or state that the review was not registered.	Page 6, lines 208–209: review protocol was not prospectively registered
	24b	Indicate where the review protocol can be accessed, or state that a protocol was not prepared.	Not reported
	24c	Describe and explain any amendments to information provided at registration or in the protocol.	**Not reported.**
Support	25	Describe sources of financial or non-financial support for the review, and the role of the funders or sponsors in the review.	Funding: page 40, lines 716–717
Competing interests	26	Declare any competing interests of review authors.	Page 1, lines 29–30; also page 39, lines 714–715
Availability of data, code and other materials	27	Report which of the following are publicly available and where they can be found: template data collection forms; data extracted from included studies; data used for all analyses; analytic code; any other materials used in the review.	Page 39, lines 711–713; page 40, lines 736–739

PRISMA 2020 Checklist

From: Page MJ, McKenzie JE, Bossuyt PM, Boutron I, Hoffmann TC, Mulrow CD, et al. The PRISMA 2020 statement: an updated guideline for reporting systematic reviews. BMJ 2021;372:n71. doi: 10.1136/bmj.n71. This work is licensed under CC BY 4.0. To view a copy of this license, visit https://creativecommons.org/licenses/by/4.0/
