# Supplementary material for: Performance, Biomechanical Demands, Injury Burden, and Health Surveillance in Wheelchair Court Sports and Selected Para Sport Cohorts: A Systematic Review
Source: Sports (Basel). 2026 Sep 7;14(9):396. doi: 10.3390/sports14090396 (PMC13611318; doi:10.3390/sports14090396)
Supplement: Supplementary file 1 [file sports-14-00396-s001.zip › Supplementary table S1.pdf]

Table S1. Quantitative Summary of Included Adaptive and Para Sport Studies (n = 22)

Study	Sport / Population	Quantitative Domain	Outcome Variable	Reported Quantitative Estimate	Statistical Finding	Interpretation
Bergamini et al. (2015) **[15]**	Junior wheelchair basketball players	Biomechanical propulsion indicators, specifically timing (push cycle duration and frequency), progression force, bilateral symmetry, and intercycle variability	The time to complete a 20-metre sprint test (*t*20*mS*), focusing on start-up and steady-state velocities	A multiple regression model showed that force- and coordination-related parameters (*PO*, *CVsym*, *CV*Δ*t*, *FCS*) accounted for 81.8% (Adjusted *R*2) of the variance in sprint time. For the experimental group, peak progression force (*Fp*) significantly increased from 330.71 ± 191.10 N to 588.32 ± 312.29 N after training	The experimental group showed statistically significant improvements in push cycle duration (*p*=0.027), frequency (*p*=0.028), progression force (*p*=0.028), and symmetry (*p*=0.046) following the training program. However, no significant difference was found for the overall *t*20*mS* time (*p*=0.080)	The training program effectively improved the "process" (propulsion technique and strength) even though the "product" (final sprint time) did not significantly change. The lack of change in total time was likely due to the high initial fitness of the athletes or the limited accuracy of manual timing. Biomechanical indicators offer valuable additional performance information for coaches beyond simple outcome scores
Cavedon et al., (2015) **[12]**	Young wheelchair basketball players	Predictors of game-related performance based on anthropometric measurements and sport-specific field test scores	Total points scored per match (TP)	A stepwise multiple regression analysis identified the following predictive model: TP = -5.589 + 0.885 (maximal pass) + 0.301 (lay-ups)	This model achieved an adjusted R^2 of 0.589 ($P < 0.001$), meaning that the combination of the maximal pass and lay-ups tests explained 58.9% of the variance in total points scored per match	Ball-handling skills—especially max pass and lay-ups—were key predictors of scoring performance. Physical factors such as age, WB experience, and fat mass % showed NS

						effect on performance. SitH2 and FC were related to success; however, the classification system mainly differentiated performance between Class A ↓ function and Class D ↑ function players.
De Witte et al. (2016) **[13]**	Wheelchair basketball Athletes	Mobility performance, quantified through time-and-motion analysis of wheelchair-athlete activities (e.g., driving forward, rotating, braking, and standing still)	Relative duration (% of absolute playtime), relative frequency (% of total activity starts), and mean duration (seconds) of specific movement activities.	National players drove forward more than international players (42.6 ± 6.8% vs. 35.4 ± 3.7%; ES = 1.48). Conversely, international players spent more time on rotational movements (28.9 ± 7.8%) compared to national players (21.8 ± 4.0%; ES = -1.30). International players also performed rotations for longer mean durations (2.3s vs. 2.1s; ES = -0.67)	Between-group factorial ANOVA identified a significant main effect for playing standard across 50% of mobility comparisons, particularly driving forward and rotation, However, there was no significant main effect of field position (guard, forward, centre) for relative duration of movement activities.	While playing standard significantly influences mobility performance—with international players demonstrating greater agility through more frequent and longer rotations—field position has minimal impact on how athletes handle their wheelchairs, this suggests that wheelchair-handling training can be largely uniform across team positions.
Iturricastillo Urteaga et al., (2016) **[36]**	Wheelchair basketball (WB) athletes	Internal match load (ML) quantification, comparing objective heart-rate (HR) methods with subjective rating-of-perceived-	The study measured objective load via Edwards ML and Stagno training impulses (TRIMPMOD), and subjective	Across 16 matches, the mean values were 255.3 ± 66.3 AU for Edwards ML, 167.9 ± 67.1 AU for TRIMPMOD, 521.9 ± 188.7 AU for sRPEres, and 536.9 ± 185.8 AU for sRPEmus.	Moderate correlations (r=.627-.668, P<.001) were found between HR-based and RPE-based methods for the whole team. However, the coefficient of	RPE-based methods are a practical, cost-effective indicator of global internal match load in WB players. However, they should not be a

		exertion (RPE) methods.	load via respiratory session RPE (sRPEres) and muscular session RPE (sRPEmus).		determination (*R*2) showed that RPE-based ML explained only ≥40% of the variance in HR-based ML. High correlations existed between the two HR methods (*r*=.959) and the two RPE methods (*r*=.919).	substitute for HR-based monitoring because they account for less than half of the variance, likely due to the high heterogeneity of physical impairments and the intermittent nature of the sport.
Van Der Slikke et al., (2016) **[49]**	wheelchair basketball athletes	Measurements focused on wheelchair kinematics (displacement, speed, acceleration), push detection via inertial sensors, and maximal isometric forward push force	Primary variables included sprint time (over a 12-meter distance), the number of pushes used to cover that distance, and maximal isometric push force	International athletes averaged a sprint time of 3.67s (SD 0.33) and 7.5 pushes (SD 1.7), while national athletes averaged 4.10s (SD 0.40) and 8.6 pushes (SD 1.0). Among national athletes, classification correlated strongly with sprint time (r = -0.715) and number of pushes (r = -0.647)	Competition level was a significant factor (p < 0.05) for all primary outcomes, whereas classification alone was not significant for the overall group. However, the interaction between competition level and classification was found to be statistically significant in General Linear Models	International level athletes perform better (faster with fewer pushes) due to superior training status and technique, even when using higher seat heights that are typically less optimal for propulsion. While classification (physical capacity) clearly dictates performance at the national level, international athletes appear to use better wheelchair configurations or advanced techniques to counteract limitations associated with lower classification.
García-Gómez &	Wheelchair	Sport-specific skills (Shooting, rebounding/one-	Shoulder Pain (SP) as measured by	27.5% of the sample reported actual SP. For	Significant main effect for gender regarding SP	Females experience significantly

Pérez-Tejero, (2017) **[28]**	basketball (WB)	handed long passes, and other sport situations).	the Shoulder Pain Index for Wheelchair Basketball (SPI-WB)	shooting and gender: $t39=2.44$ with a moderate effect size (Cohen's d).	during shooting skills ($p \leq 0.05$). No significant differences for age or other skills.	more shoulder pain than males during shooting, suggesting that shoulder pain affects performance differently based on gender.
Wilroy & Hibberd (2018) **[30]**	collegiate wheelchair basketball athletes	The researchers focused on the shoulder range of motion (ROM) and shoulder/scapular muscle strength	The primary measures were dominant limb internal rotation (IR) ROM, external rotation (ER) ROM, IR strength, ER strength, and scapular retraction strength	IR ROM: Increased by an average of 11.4° (Pre: 31.00° ± 3.76°; Post: 42.43° ± 7.02°). ER ROM: Increased by an average of 8.0° (Pre: 92.02° ± 11.59°; Post: 100.00° ± 10.20°). IR Strength: Pre: 27.08 ± 11.07 lbs; Post: 46.05 ± 39.68 lbs. ER Strength: Pre: 24.29 ± 10.24 lbs; Post: 22.92 ± 8.61 lbs. Retraction Strength: Pre: 18.40 ± 7.14 lbs; Post: 17.80 ± 7.14 lbs.	There were statistically significant improvements in dominant limb shoulder IR ROM ($t6=3.56, p=0.012$) and ER ROM ($t6=2.79, p=0.032$). There were no significant increases in shoulder IR strength, ER strength, or scapular retraction strength ($p>0.05$).	The sources suggest that a 6-week strengthening and stretching intervention can effectively improve shoulder ROM in wheelchair basketball athletes, which is considered clinically significant and may decrease risk factors for shoulder injury. The lack of significant strength gains may be due to the participants' high baseline activity levels (team practices, daily wheelchair propulsion, and weight training), which likely made the resistance-tubing program insufficiently robust to create further strength improvements.
Rietveld et al., 2019) **[21]**; Netherlands	Wheelchair tennis players	Assessment of inter-trial reliability and construct validity of four sport-specific field tests using	Key variables included end times (s), mean/peak linear velocity (m/s), peak forward	Inter-trial reliability for end times showed Intraclass Correlation Coefficients (ICCs) of 0.95-	Significant differences ($p<0.05$) were found between groups for almost all kinematic variables across	The four field tests are reliable and valid for evaluating mobility in elite wheelchair

		Inertial Measurement Units (IMUs).	acceleration (m/s²), mean/peak rotational velocity (°/s), and peak rotational acceleration (°/s²).	0.99. For construct validity, the adult group was significantly faster than juniors, such as in the 20m Sprint (5.90s vs. 7.27s) and the Spider test (16.18s vs. 19.19s).	the 20m Sprint, Spider, and Illinois tests. However, the Illinois test showed a systematic learning effect between trials (*p*=0.02), with trial 2 being faster (21.04s) than trial 1 (21.33s).	tennis. Because the Spider, Butterfly-sprint, and Illinois tests are highly correlated (*r*>0.75), the authors recommend using only the 20m Sprint for sprinting and the Spider test for manoeuvrability to save time while maintaining performance insights.
Fagher et al., (2020) **[39]**	Swedish Paralympic athletes	Annual incidence, severity, and risk factors for sports-related injuries and illnesses (SRIIPS)	Annual Incidence Proportion (IP), Incidence Rate (IR) per 1000 hours of exposure, and Hazard Ratios (HR) for identified risk factors	Annual IP: 68% for injuries; 77% for illnesses. Incidence Rate: Injury IR of 6.9/1000 hours; Illness IR of 9.3/1000 hours. Median Time to Event: 19 weeks for first injury; 9 weeks for first illness. Injury Risk (HR): Team sports (1.88), previous severe injury (2.37), and male athletes (1.76). Illness Risk (HR): Team sports (1.64) and males with a previous illness (2.13).	Significant variations ($p < .05$) were found in injury survival probability regarding gender, sport type, and previous injury history. Additionally, athletes with a middle training load reported a significantly higher illness IR (22/1000 hours; $p = 0.019$).	Paralympic athletes face a high burden of injury and illness, often exceeding rates seen in able-bodied athletes. Because the majority of injuries occur during training rather than competition, there is a critical need for year-round, individualized preventive strategies and specialized medical support for this population.
Franchin et al., (2020) **[17]**	National Wheelchair Rugby Team Athletes	Propulsion kinematics and biomechanical characteristics during sports-specific dynamic tests on an inertial drum bench	Elbow Range of Motion (ROM); Contact, Release, and Total Push Angles; and the percentage of time spent	In the sprint test, overall elbow ROM increased from 68.4° ± 14.3° at the start to 76° ± 10.98° at the end. The mean percentage of time spent in the	Speed increases in sprinting were achieved through a mean increment in release angles of 8.5° ± 3.7° and a mean reduction in contact angles of 9.1° ± 5.2°,	Most athletes use a semi-circular propulsion pattern, which is considered protective for the upper limbs.

			in the push phase	push phase during the Wingate test was 50.1%, compared to 44.4% during the sprint test.	effectively displacing the total push angle forward.	However, the requirement to increase velocity leads to more anteriorly displaced push angles, a factor that increases mechanical stress and loading on the shoulder joint, potentially leading to overuse injuries.
Ju et al. (2021) **[20]**	Wheelchair (WC) tennis	Kinematics (trunk and shoulder angular position, excursion, velocity, and acceleration) and impact vibration (vibration intensity over time).	Trunk rotation angular position at the start of the preparation phase (AP-PS) and Racket vibration intensity (VI).	Trunk Rotation (AP-PS): Standing AB players = 10.8 ± 6.2°; Sitting AB players = 46.6 ± 18.1°; WC players = 42.3 ± 17.2°. Racket Vibration Intensity (VI): Standing AB players = 4949.69 ± 1107.75 g*s; Sitting AB players = 4949.52 ± 116.92 g*s; WC players = 3894.33 ± 585.84 g*s.	For trunk rotation (AP-PS), there were significant differences between standing players and both sitting AB and WC players ($p < 0.001$), but no significant difference between sitting AB and WC players ($p = 0.516$). The racket vibration was significantly lower for WC players compared to both standing and sitting AB players ($p = 0.006$).	Because WC tennis players lack the power generated by the lower limbs and trunk, they employ "biomechanical solutions" such as reinforced trunk rotation before hitting the ball. This creates a stretch-shortening cycle that accumulates elastic potential energy to compensate for the incomplete kinetic chain. Additionally, while WC players experience lower racket vibration, they have a lower vibration reduction ratio (42.86%) from the racket to the wrist compared to AB players, suggesting their vibration

						absorption is less efficient, which may increase the risk of injuries like tennis elbow.
Demeco et al., (2022) **[29]**	male professional wheelchair basketball athletes	Functional Status & Pain	Wheelchair User's Shoulder Pain Index (WUSPI)	Baseline (T0): 27.0 ± 18.5 vs. post-intervention (T1): 25.0 ± 21.5	Significant improvement (p = 0.009).	The program reduced pain levels experienced during daily functional activities
Fapojuwo et al., (2022) **[27]**	wheelchair basketball athletes	Epidemiological cross-sectional survey focusing on injury prevalence, patterns, and perceived risk factors	The primary variables measured were career prevalence, 12-month prevalence, and point prevalence of injuries, along with specific anatomical sites and perceived risk factors	Career prevalence: 93.7%. 12-month prevalence: 61.9%. Point prevalence: 3.2%. Most common injury site: Shoulder (66.7%), followed by the forearm (31.7%) and wrist/hand (30.2%). Perceived risk factors: Fatigue/overwork (30.2%) and contact with other athletes (28.6%)	There were no significant associations ($p \geq 0.05$) found between the 12-month injury prevalence and the participants' age (p=0.72), sex (p=0.27), or duration of sports participation (p=0.37). The association between finger injuries and the 12-month prevalence was assessed as close to statistical significance (p=0.05)	The study indicates a very high prevalence of musculoskeletal injuries in this population, significantly higher than some reported rates in other countries. The upper extremities are uniquely vulnerable because athletes use them continuously for both high-intensity sports propulsion and daily mobility. Contributing environmental and economic factors include training on concrete surfaces (68.3%) and a lack of protective gear (61.9%) due to financial constraints or the perception that it is unnecessary.

Haydon et al., (2022) **[50]**	Wheelchair Rugby (WCR) elite players	Biomechanics and predictive modelling, specifically utilizing a two-step approach consisting of a linkage model for propulsion kinematics and partial least-squares (PLS) regression for performance.	The primary outcome variables were propulsion kinematics (hand contact and release angles for the first three strokes) and sprint performance (time for a 5m sprint).	For propulsion kinematics, mean prediction errors for contact angles were less than 5 degrees for 43 of 48 predictions. Performance prediction results showed a mean sprint time error of 0.04 ± 0.25 seconds across all players, with a minimum error of 0.01 s for one player and a maximum of 0.87 s for another.	There were no significant differences in contact angle prediction accuracy between the first three strokes. However, release angle prediction errors were significantly lower for the first stroke compared to the second ($p < 0.001$) and third strokes ($p < 0.001$). While six of the eight players had average performance prediction errors of less than 0.1 seconds, results for two players (Players 5 and 7) were markedly less accurate.	The proposed modeling approach shows potential for identifying trends in how changes to wheelchair setup (such as seat height or angle) affect performance, but its accuracy is currently inconsistent across different athletes. The researchers suggest that while a simplified linkage model may work for some, more complex musculoskeletal models may be necessary to account for individual activity limitations and functional variations to improve prediction reliability.
Alim et al., (2023) **[18]**	Wheelchair tennis (WT)	Neuromuscular and physical field tests focusing on strength, speed, agility, and endurance	Serve accuracy, measured using the Hewitt Service Test.	Medicine Ball Throw (MBT) Serve: $r = 0.874$ ($p = 0.001$).<br>MBT Forehand: $r = 0.811$ ($p = 0.004$).<br>MBT Backhand: $r = 0.747$ ($p = 0.013$).<br>Sprint 20m with a racket: $r = -0.718$ ($p = 0.019$).<br>Logistic Regression (MBT Group): Nagelkerke R	MBT-serve ($r = 0.874$, $p = 0.001$), MBT-forehand ($r = 0.811$, $p = 0.004$), and MBT-backhand ($r = 0.747$, $p = 0.013$) were positively associated with serve accuracy. Sprint 20m with racket was negatively associated with serve accuracy ($r = -0.718$, $p = 0.019$), indicating	Upper body explosive strength and rotational power in the trunk are the primary physical determinants of serve accuracy in wheelchair tennis. Because wheelchair athletes have limited lower-body

				Square = 1.00 with a 100% correct classification rate for accuracy levels.	faster sprint time → better accuracy. Grip strength, agility, and endurance were non-significant ($p > 0.05$). MBT variables showed strong prediction of serve accuracy level (Nagelkerke $R^2 = 1.00$; 100% classification).	involvement in the kinetic chain, they must rely heavily on trunk rotation and upper extremity power to generate the stability and force necessary for accurate service placement.
Briley et al., (2023) **[16]**	Elite Wheelchair Rugby players	Biomechanical adaptations to fatigue (kinetics and kinematics of sprinting)	Sprint Performance (Peak Velocity and Distance) and Propulsion Biomechanics (Thorax/Shoulder kinematics and Asymmetries)	Vmax (Overall): Pre-ISP 3.79 ± 0.62 vs. post-ISP 3.79 ± 0.59 $m \cdot s^{-1}$; Thorax Flexion: decreased -5° (acceleration) and -6° (max velocity); GH Abduction Asymmetry: +20.2% (max velocity); SCI Peak Power Asymmetry: +6.9%	No significant change in sprint peak velocity or distance ($p > 0.05$) despite significant increases in physiological markers (HR, BLa, RPE). Significant main effects for time ($p < 0.05$) were found for reduced thorax flexion, peak GH abduction, and increased asymmetries.	Elite players maintain sprint performance despite physiological fatigue by modifying their propulsion technique. Reducing shoulder abduction may be a protective response to minimize acute stress or perceived threat of pain at the shoulder.
Esak et al. (2024) **[19]**	Wheelchair tennis athletes	Biomechanical propulsion dynamics and wheelchair manoeuvrability.	Propulsion Velocity (m/s)	$r = 0.840$ for the correlation between push frequency and velocity; $r = -0.859$ for the correlation between stroke time and velocity.	$p < 0.001$ for both primary correlations, indicating high statistical significance.	Wheelchair manoeuvrability is directly enhanced by a higher push frequency (more pushes per second) and a shorter stroke time (reduced duration of a single push cycle). Elite athletes demonstrate superior manoeuvrability compared to novices by maintaining

						higher push frequencies and higher mean velocities. Additionally, holding a racquet generally reduces maximal propulsion speed and increases the Rate of Perceived Exertion (RPE).
Hernández-Beltrán et al.,(2023) **[51]**	Wheelchair Basketball (WB)	The research analyzed External Load (EL) and Internal Load (IL) demands. External load was quantified through kinematics and neuromuscular variables using inertial devices, while internal load was measured via heart rate (HR) monitors	Kinematics External Load: Distance/min, explosive distance/min, acceleration (Acc)/min, deceleration (Dcc)/min, high-speed running (HSR)/min, maximum Acc, maximum Dcc, average speed, and maximum speed. Neuromuscular External Load: Player load (PL)/min and impacts/min. Objective Internal Load: Average HR (bpm), maximum HR (bpm), and % maximum HR.	Distance/min: 63.43 meters in training vs. 95.48 meters in matches. Acc/min: 26.26 m/s^2 in training vs. 75.35 m/s^2 in matches. Dcc/min: 22.39 m/s^2 in training vs. 75.80 m/s^2 in matches. Average HR: 118.92 bpm in training vs. 133.83 bpm in matches. % Maximum HR: 68.44% in training vs. 69.49% in matches.	Significant differences ($p < 0.05$) were found between training and competition for 13 out of 14 variables, with matches showing higher values for almost all metrics except HSR/min and maximum/average speeds. Deceleration per minute (Dcc/min) reported the highest Effect Size (2.237), followed by acceleration per minute (2.092), indicating these variables had the most pronounced differences between contexts. % Maximum HR was the only variable that did not present a significant difference ($p = 0.556$) between training and matches. Players with a higher functional classification (FC 4.0) showed significant differences in	Official matches are significantly more physically and physiologically demanding than 5x5 training situations in wheelchair basketball. The high number of accelerations and decelerations in competition highlights the intermittent nature of the sport. Furthermore, players with higher functional classifications (FC 4.0) achieve greater performance values due to their increased trunk stability and range of movement. The authors conclude that coaching staff must design

					kinematic and neuromuscular EL variables compared to lower classifications	training sessions that are more intensive and explosive to better simulate the specific demands of competition and reduce the risk of injury from overload
Hernández-Beltrán et al. (2024) **[14]**	professional national wheelchair basketball (WB) players	Analysis of External Load (EL), including kinematic and neuromuscular variables, and Internal Load (IL), focusing on physiological responses	Primary variables included distance covered per minute, average/maximum speed, accelerations/decelerations, player load, total impacts, and heart rate (HR) metrics.	Players with the highest functional classification (FC 4.0) recorded a distance of 54.76 ± 20.12 m/min, a player load of 0.71 ± 0.27 a.u./min, and a maximum HR of 172.83 ± 18.01 bpm. In contrast, FC 1.0 players recorded 35.61 ± 15.60 m/min for distance and 150.92 ± 27.13 bpm for maximum HR.	Significant differences ($p < 0.05$) were identified between functional classes, with FC 4.0 players showing higher values in distance, decelerations, average speed, player load, impacts, and heart rate compared to other groups. Correlational analysis also showed that height and experience positively influence average heart rate and maximum speed.	Players with higher functional capacity (FC 4.0) exhibit superior physical and movement capabilities due to better trunk control and a larger range of motion. This data suggests that coaching staff must individualize training loads and task designs based on a player's functional classification to maximize performance and reduce injury risk from overexertion.
Busch et al., (2025) **[22]**	German Paralympic squad athletes	Epidemiology and risk factor analysis for health problems (injuries and illnesses) over a 124-week longitudinal observation period	Average weekly prevalence, incidence rates, and hazard/odds ratios for substantial health problems (those causing a reduction in performance or sports absence)	The cohort reported 438 total health problems (51% illnesses, 49% injuries). The average weekly prevalence for substantial health problems was 10.5% (95% CI: 9.6-11.5%). New acute injuries peaked at 31.8% during competition weeks, while new overuse injuries peaked at 41.2%	Females had an 80% higher risk (Hazard Ratio = 1.8; 95% CI: 1.2-2.8) of sustaining a substantial health problem compared to males. Every additional year of elite training experience was associated with a lower risk of substantial health problems (Odds Ratio = 0.9; 95% CI: 0.8-0.9). Higher than	Female athletes and novices (those with <5 years of experience) require closer monitoring and targeted prevention strategies. Seasonal periodization significantly influences injury types, with training camps being a

				during training camps	normal training intensity was also a significant risk factor (OR = 1.6; 95% CI: 1.2-1.9)	high-risk period for overuse issues and competitions for acute injuries
Fallon et al., (2025) **[32]**	profession al endurance Para cyclists	Prospective epidemiology focused on the incidence, burden, and severity of injuries and illnesses, alongside subjective well-being metrics	Incidence rates of self-reported and medical attention health problems, injuries, and illnesses (expressed per 365 days and per 1000 h), as well as subjective markers of fatigue, mood, stress, and sleep quality	Athletes reported 7.36 health problems per 365 days (95% CI: 5.41-9.46).<br>The medical attention rate was 5.56 per 365 days (95% CI: 3.91-7.36).<br>The overall injury rate was 1.94 per 365 athlete days (Male: 2.44; Female: 1.51).<br>The overall illness rate was 3.60 per 365 days (Male: 1.80; Female: 5.40).<br>The shoulder was the most common injury location (0.60 per 365 days), and respiratory illness was the most prevalent illness (56.0% of cases).	Multivariable Poisson regression found that athletes with a health problem reported significantly higher levels of perceived fatigue ($p < 0.001$).<br>Female athletes were over three times more likely to experience respiratory illnesses than males (RR 3.66, $p \leq 0.05$).<br>A two-sample Poisson test showed no significant difference between the reporting rate of the OSTRC-H2 questionnaire and the medical log ($p = 0.235$).<br>Associations between health problems and mood ($p = 0.088$), sleep ($p = 0.134$), or stress ($p = 0.223$) were not statistically significant	The study demonstrates that longitudinal health monitoring is feasible and effective in Para-cycling for tracking both physical and mental well-being. The findings suggest a distinct sex difference in health risks, with males more prone to injury and females significantly more susceptible to illness, particularly respiratory infections. This highlights the need for proactive, multidisciplinar y support systems to manage fluctuations in athlete well-being during the Paralympic season.
van der Slikke et al. (2026) **[10]**	wheelchair tennis (WT) players,	Analysis of the relationship between trunk/arm strength and wheelchair mobility performance during standardized field tests and elite match play	Maximal speed, average acceleration, and maximal/avera ge rotational speed and acceleration captured via Inertial Measurement Units (IMUs).	Athletes with full trunk control (Trunk 2) reached 4.1 ± 0.4 m/s maximal match speed compared to 3.2 ± 0.5 m/s for those with non-functional trunks (Trunk 0). Effect sizes for mobility differences between these groups were large,	Significant differences ($p < 0.01$) were found across trunk function groups for speed, acceleration, and rotation. Conversely, correlations between isometric arm strength and mobility were only modest ($r = 0.26$-0.62).	Trunk function is a critical determinant of WT mobility (especially rotation and acceleration) but is underrepresente d in the current classification system, where it accounts for only 14% of the total score.

	ranging from 1.18 to 2.43.	Classification should prioritize functional, sport-specific measures like dynamic trunk movement over static strength assessments.

Note- *WT = Wheelchair Tennis; WB = Wheelchair Basketball; WCR = Wheelchair Rugby; WC = Wheelchair; AB = Able-bodied; SCI = Spinal Cord Injury; ALD = Amputation/Limb Difference; MBT = Medicine Ball Throw; SA = Serve Accuracy; IMU = Inertial Measurement Unit; ICC = Intraclass Correlation Coefficient; SEM = Standard Error of Measurement; AP-PS = Acceleration/Preparation Phase to Preparation Start; VI = Vibration Intensity; SP = Shoulder Pain; SPI-WB = Shoulder Pain Index for Wheelchair Basketball; ML = Match Load; HR = Heart Rate; RPE = Rating of Perceived Exertion; sRPE = Session Rating of Perceived Exertion; sRPEres = Respiratory Session Rating of Perceived Exertion; sRPEmus = Muscular Session Rating of Perceived Exertion; TRIMP = Training Impulse; TRIMPMOD = Modified Training Impulse; AU = Arbitrary Units; ROM = Range of Motion; IR = Internal Rotation; ER = External Rotation; PLS = Partial Least Squares; ES = Effect Size; EL = External Load; IL = Internal Load; Acc = Acceleration; Dcc = Deceleration; HSR = High-Speed Running; PL = Player Load; FC = Functional Classification; WUSPI = Wheelchair User's Shoulder Pain Index; SRIIPS = Sports-Related Injuries and Illnesses in Paralympic Sport; IP = Incidence Proportion; IR = Incidence Rate; RR = Relative Risk/Risk Ratio; OR = Odds Ratio; CI = Confidence Interval; BLa = Blood Lactate; GH = Glenohumeral; t20mS = Time to Complete 20-Metre Sprint; TP = Total Points Scored per Match; Fp = Peak Progression Force; CVsym = Coefficient of Variation of Symmetry; CVΔt = Coefficient of Variation of Time Difference; FCS = Functional Classification Score; OSTRC-H2 = Oslo Sports Trauma Research Center Questionnaire on Health Problems, version 2; p = Probability Value; r = Correlation Coefficient;* R^2 *= Coefficient of Determination; Adjusted* R^2 *= Adjusted Coefficient of Determination; t = t-test Statistic; n = Sample Size; SD = Standard Deviation.*
